# Baseline Low-Density-Lipoprotein Cholesterol Modifies the Risk of All-Cause Death Associated With Elevated Lipoprotein(a) in Coronary Artery Disease Patients

**DOI:** 10.3389/fcvm.2021.817442

**Published:** 2022-01-13

**Authors:** Younan Yao, Jin Liu, Bo Wang, Ziyou Zhou, Xiaozhao Lu, Zhidong Huang, Jingru Deng, Yongquan Yang, Ning Tan, Shiqun Chen, Jiyan Chen, Yong Liu

**Affiliations:** ^1^Department of Cardiology, Guangdong Cardiovascular Institute, Guangdong Provincial People's Hospital, Guangdong Academy of Medical Sciences, Guangzhou, China; ^2^Guangdong Provincial Key Laboratory of Coronary Heart Disease Prevention, Guangdong Cardiovascular Institute, Guangdong Provincial People's Hospital, Guangdong Academy of Medical Sciences, Guangzhou, China; ^3^School of Medicine, Guangdong Provincial People's Hospital, South China University of Technology, Guangzhou, China; ^4^The Second School of Clinical Medicine, Southern Medical University, Guangzhou, China

**Keywords:** coronary artery disease, statin, lipoprotein(a), low-density-lipoprotein cholesterol, all-cause mortality

## Abstract

**Background:** The prognostic value of elevated lipoprotein(a) [Lp(a)] in coronary artery disease (CAD) patients is inconsistent in previous studies, and whether such value changes at different low-density-lipoprotein cholesterol (LDL-C) levels is unclear.

**Methods and Findings:** CAD patients treated with statin therapy from January 2007 to December 2018 in the Guangdong Provincial People's Hospital (NCT04407936) were consecutively enrolled. Individuals were categorized according to the baseline LDL-C at cut-off of 70 and 100 mg/dL. The primary outcome was 5-year all-cause death. Multivariate Cox proportional models and penalized spline analyses were used to evaluate the association between Lp(a) and all-cause mortality. Among 30,908 patients, the mean age was 63.1 ± 10.7 years, and 76.7% were men. A total of 2,383 (7.7%) patients died at 5-year follow-up. Compared with Lp(a) <50 mg/dL, Lp(a) ≥ 50 mg/dL predicted higher all-cause mortality (multivariable adjusted HR = 1.19, 95% CI 1.07–1.31) in the total cohort. However, when analyzed within each LDL-C category, there was no significant association between Lp(a) ≥ 50 mg/dL and higher all-cause mortality unless the baseline LDL-C was ≥ 100 mg/dL (HR = 1.19, 95% CI 1.04–1.36). The results from penalized spline analyses were robust.

**Conclusions:** In statin-treated CAD patients, elevated Lp(a) was associated with increased risks of all-cause death, and such an association was modified by the baseline LDL-C levels. Patients with Lp(a) ≥ 50 mg/dL had higher long-term risks of all-cause death compared with those with Lp(a) <50 mg/dL only when their baseline LDL-C was ≥ 100 mg/dL.

## Introduction

The convincing evidence from epidemiological and Mendelian randomization studies indicated that elevated lipoprotein(a) [Lp(a)] was causally associated with incident atherosclerotic cardiovascular diseases in the general population (1–5). Multiple pathways were believed to contribute to this low-density-lipoprotein-like particle's pathogenic effects, including pro-atherogenic, pro-thrombotic, and pro-oxidative properties (6). However, because no drug is proved to specifically decrease serum Lp(a) with subsequent adverse events such as major adverse cardiovascular events (MACEs) by randomized controlled trials, Lp(a) is only recommended to be used in risk stratification by the last European and American cholesterol management guidelines (7, 8).

Recent studies found that compared with the primary preventive population, the estimated per mg/dL relative risk reduction of Lp(a) in adverse events in patients with post-acute coronary syndrome (ACS) was greater, which indicated that Lp(a) reduction might potentially generate more benefits in the secondary prevention setting (2, 9). However, the association between elevated Lp(a) and higher risk of adverse events in the secondary preventive population was inconsistent in previous studies (9–16). A *post-hoc* analysis of the ODYSSEY OUTCOMES (ODYSSEY Outcomes: Evaluation of Cardiovascular Outcomes After an Acute Coronary Syndrome During Treatment with Alirocumab) trial found that baseline Lp(a) predicted the risk of MACE after recent ACS (9). By contrast, the reanalyses of the dal-Outcomes trial and the SATURN study (Study of Coronary Atheroma by Intravascular Ultrasound: Effect of Rosuvastatin vs. Atorvastatinand) showed that Lp(a) concentration was not associated with adverse cardiovascular outcomes (13). Furthermore, it had been suggested that significant associations between Lp(a) and outcomes might depend on concurrent high levels of low-density-lipoprotein cholesterol (LDL-C) (11, 12, 15). A study in a primary prevention setting also found that the risk was associated with elevated Lp(a) attenuated if LDL-C was <2.5 mmol/L (5). However, whether the prognostic value of elevated Lp(a) in patients with established coronary artery disease (CAD) would be modified by different baseline LDL-C levels had not been systematically investigated. With the cholesterol-lowering therapies such as statins and the addition of either ezetimibe or monoclonal antibodies against proprotein convertase subtilisin/kexin type 9 (PCSK9), LDL-C could be efficiently controlled to a very low level of <70 mg/dL or even <55 mg/dL. Therefore, it is critical for us to identify potential CAD patients who may benefit (or benefit more) from Lp(a)-lowering agents if specific therapy is available in future clinical practice. In this study, we hypothesized that elevated Lp(a) in CAD patients is associated with increased risks of all-cause death, and such an association can be modified by their baseline LDL-C levels.

## Methods

### Participants

This study was based on the Cardiorenal ImprovemeNt (CIN) registry database (NCT04407936). Briefly, the CIN registry study was a retrospective cohort study which was designed to explore the risk factors and prognosis of adverse kidney events in patients undergoing coronary angiography from January 2007 to December 2018 in the Guangdong Provincial People's Hospital. Patients diagnosed with CAD according to the 10th Revision Codes of the International Classification of Disease (ICD-10; I20-I25, I50.00001 and I91.40001) and with at least one major coronary artery stenosis ≥ 50% by coronary angiography were consecutively recruited in this study. We excluded patients: (1) <18 years; (2) dying during hospitalization or discharging automatically (who refused to die in the hospital); (3) suffering from any tumor; (4) with chronic obstructive pulmonary disease; (5) discharging without statin treatment; (6) without the data of estimated glomerular filtration rate (eGFR); (7) with eGFR <30 mL/min/1.73m^2^ or treated with dialysis; (8) without baseline Lp(a) and LDL-C; (9) without follow-up data. Because PCSK9 inhibitors were not introduced in the Guangdong Provincial People's Hospital until 2019, all patients in our study were not prescribed with PCSK9 inhibitors at discharge. This study was approved by the Guangdong Provincial People's Hospital Ethics Committee and was performed according to the Declaration of Helsinki.

### Data Collection

Baseline data, including demographic characteristics, comorbidities, laboratory examinations, in-hospital treatments, and medications prescribed at discharge, were extracted from the electronic clinical management system of the Guangdong Provincial People's Hospital. Comorbidities were determined by the diagnosis before admission or for the first-time during hospitalization. Missing values were treated by using multiple imputation with chained equations (see Supplementary Material and Supplementary Table 1).

Baseline lipid assessments were performed at admission. Lp(a) mass was evaluated by an auto immunoturbidimetry assay on a chemistry analyzer (AU5800 Analyzer, Beckman Coulter, Brea, California). The intraassay coefficient of variation was ≤ 4% and the interassay coefficient of variation was ≤ 10%. LDL-C was assessed by a direct assay on the same chemistry analyzer. The intraassay coefficient of variation was ≤ 3% and the interassay coefficient of variation was ≤ 4%. LDL-C was converted from mmol/L to mg/dL (by multiple by 38.67) (17). Because the clinical measure of LDL-C includes the cholesterol content of Lp(a), which contributes approximately 30% of Lp(a) mass (18), we calculated corrected LDL-C (LDL-Ccorr) using the following formula (18):


$$\begin{array}{l} \text{LDL}-\text{Ccorr}\left[\text{mg}/\text{dL}\right]=\text{LDL}-\text{C}\left[\text{mg}/\text{dL}\right]\\ \;-0.3\;*\;\mathrm{Lp}\left(\text{a}\right)\;\left[\mathrm{mg}/\mathrm{dL}\right]. \end{array}$$

### Follow-Up and Outcome

The primary outcome of this study was all-cause death at 5-year follow-up (long term). The secondary outcomes were all-cause death at 1-year (short term) and 3-year (medium term) follow-up. Data on all-cause death and follow-up time were obtained from the Guangdong Provincial Public Security and were matched to the electronic clinical management system of the Guangdong Provincial People's Hospital according to patients' resident ID numbers. Survival time was calculated from the date of discharge. In order to calculate the hazard ratios of all-cause death at 1-, 3-, and 5-year follow-up, we censored survival time if patients didn't experience death before the corresponding time points.

### Statistical Analysis

Patients were divided into groups with Lp(a) <50 and ≥50 mg/dL. Cut-offs for LDL-C were set at 70 and 100 mg/dL according to the 2016 European Guidelines on cardiovascular disease prevention in clinical practice (19), dividing patients into groups with LDL-C <70, 70– <100, and ≥100 mg/dL, respectively. Baseline characteristics were compared between Lp(a) groups. Continuous variables were reported as mean ± SD or median (25^th^-75th percentile) and compared by the *t*-test or Wilcoxon-rank test as appropriate. Categorical variables were displayed as percentages (number) and compared by the Chi-square test. Baseline characteristics were also compared across LDL-C categories as well as between Lp(a) groups by LDL-C categories (Supplementary Material). Correlations of Lp(a) with other lipid parameters were assessed by the Spearman correlation test.

The Kaplan-Meier survival curves and the complementary log-log (Cloglog) tests for fixed time points (20) were used to determine the cumulative survival at 1-, 3-, and 5-year follow-up among patients with different Lp(a) levels. To explore the association between baseline Lp(a) and all-cause mortality in the total cohort and three LDL-C groups, Cox proportion hazard analyses were performed to calculate hazard ratios (HRs) with 95% confident intervals (CIs). The proportional hazard assumption was examined by the inspection of Schoenfeld residuals. We used univariate Cox regression models to compute crude HRs and 95% CIs. In multivariate analyses, a backward stepwise method according to minimal Akaike Information Criterion (AIC), with all baseline variables entered, was used to identify the variables associated with all-cause mortality. Given the number of the events available in patients whose LDL-C was <70 mg/dL, covariates in full adjustment models were carefully selected with additional regards for clinical considerations (Supplementary Material). In the end, the following covariates were included: age, gender, congestive heart failure (CHF), hypertension, diabetes mellitus, percutaneous coronary intervention (PCI) or coronary artery bypass graft (CABG), eGFR, high-density-lipoprotein cholesterol (HDL-C), and triglyceride. To evaluate the potential non-linear association between Lp(a) levels and all-cause mortality, penalized spline analyses were used by us with the same covariates aforementioned. Considering the potential variation of LDL-Ccorr within each LDL-C group, we additionally adjusted for LDL-Ccorr to assess whether the association between Lp(a) and all-cause death would change. The prognostic value modification of Lp(a) was assessed *via* the introduction of the interaction terms between Lp(a) groups and LDL-C (continuous or categorical) in the total cohort.

Additionally, we calculated multivariate-adjusted HRs for Lp(a) using the threshold of 30 mg/dL. Sensitivity analyses were also performed in patients without acute myocardial infarction (AMI) as well as in those undergoing PCI or CABG.

All analyses were conducted by using R software version 4.0.1. Due to the exploratory nature of these analyses, no adjustments were made for multiple comparisons. *P* < 0.05 were considered statistically significant.

## Results

### Baseline Characteristics

**Figure 1:**
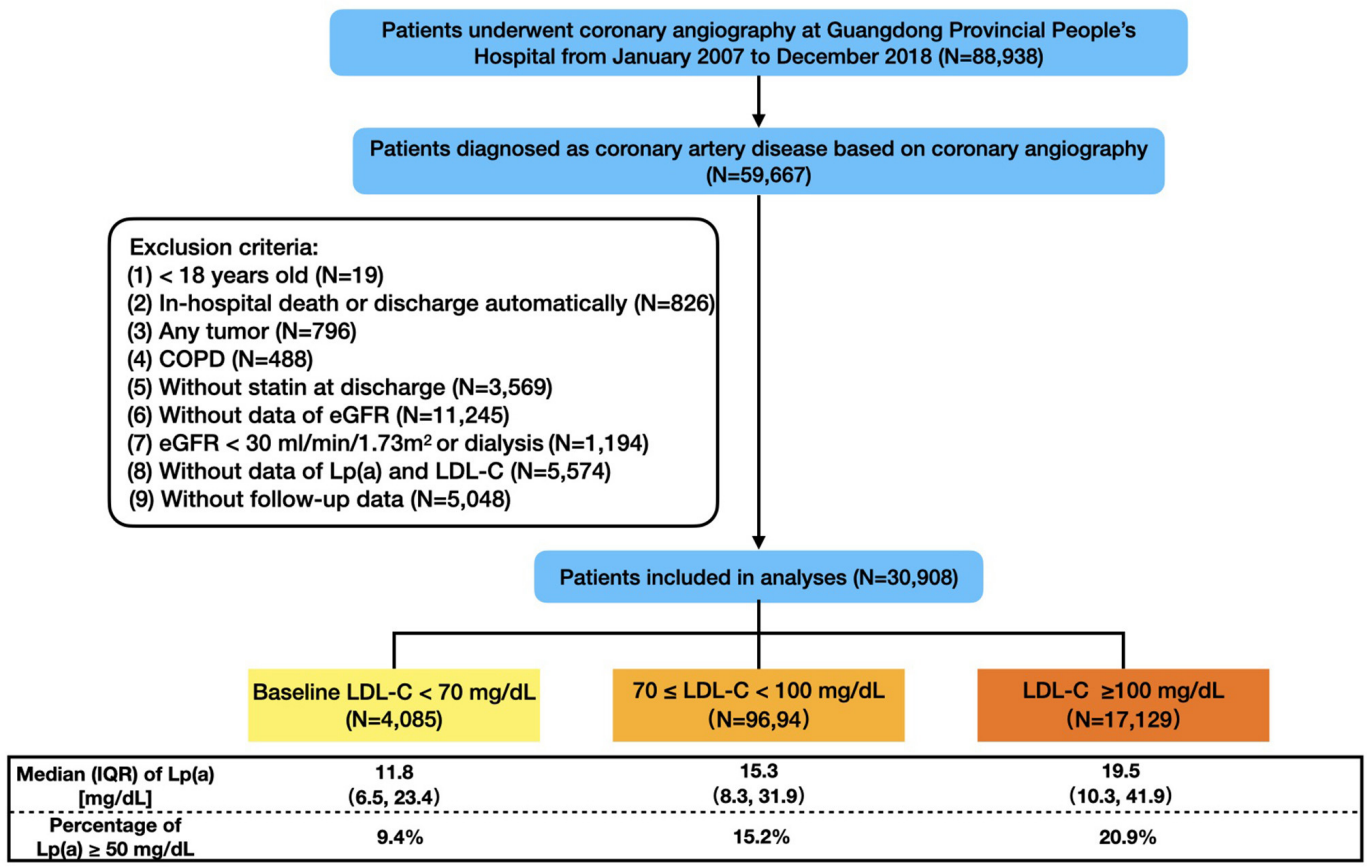
Flowchart of study population. COPD, chronic obstructive pulmonary disease; eGFR, estimated glomerular filtration rate; LDL-C, low-density-lipoprotein cholesterol; Lp(a), lipoprotein (a); IQR, interquartile range.

The flowchart was shown in Figure 1. In the end, 30,908 patients were included in this study, and the baseline characteristics were displayed in Table 1. The mean age was 63.1 ± 10.7 years and 76.7% were male. Compared with the patients in Lp(a) <50 mg/dL group, those in Lp(a) ≥ 50 mg/dL group were more likely to have chronic kidney disease, AMI, CHF, and stroke but were less frequent to suffer from hypertension, diabetes mellitus, and atrial fibrillation. The median levels of Lp(a) were 13.5 (7.9–23.2) and 81.3 (62.4–107.3) mg/dL for individuals with an Lp(a) <50 and ≥ 50 mg/dL, respectively. The patients in Lp(a) group of ≥50 mg/dL had higher levels of total cholesterol, LDL-C, non-HDL-C, and apoprotein B; however, the serum concentrations of LDL-Ccorr and triglyceride were lower in Lp(a) group of ≥50 mg/dL. Additionally, individuals with Lp(a) ≥ 50 mg/dL were more likely to receive revascularization treatment, angiotensin converting enzyme inhibitor/angiotensin receptor blocker, beta blocker, and anti-platelet drugs.

**Table 1:** Baseline characteristics of patients by levels of Lp(a).

	**Total cohort** **(*n* = 30,908)**	**Lp(a) <50 mg/dL** **(*n* = 25,475)**	**Lp(a) ≥50 mg/dL** **(*n* = 5,433)**	***P*-value**
**Demographics**				
Age, years	63.1 ± 10.7	63.2 ± 10.7	62.7 ± 10.5	0.002
Male, (%)	76.7% (23,717)	77.2% (19,666)	74.6% (4,051)	<0.001
**Comorbidities**				
Hypertension, (%)	56.9% (17,593)	57.3% (14,594)	55.2% (2,999)	0.005
Diabetes, (%)	27.8% (8,602)	28.4% (7,235)	25.2% (1,367)	<0.001
CKD, (%)	18.6% (5,747)	18.0% (4,584)	21.4% (1,163)	<0.001
AMI, (%)	21.5% (6,637)	21.0% (5,342)	23.8% (1,295)	<0.001
CHF, (%)	9.1% (2,815)	8.8% (2,253)	10.3% (562)	0.001
Stroke, (%)	5.8% (1,804)	5.7% (1,453)	6.5% (351)	0.033
AF, (%)	2.8% (858)	2.9% (746)	2.1% (112)	<0.001
**Laboratory measurement**				
Hemoglobin, g/L	134 ± 16	134 ± 16	132 ± 16	<0.001
eGFR, ml/min/1.73m^2^	79.8 ± 22.5	80.1 ± 22.4	78.3 ± 22.9	<0.001
TC, mmol/L	4.40 (3.70, 5.20)	4.33 (3.65, 5.16)	4.62 (3.92, 5.47)	<0.001
LDL-C, mg/dL	104.4 (82.8, 130.3)	102.5 (81.2, 128.0)	113.3 (91.7, 140.0)	<0.001
**LDL-C categories**				<0.001
<70 mg/dL	13.2% (4,085)	14.5% (3,700)	7.1% (385)	
70– <100 mg/dL	31.4% (9,694)	32.3% (8,217)	27.2% (1,477)	
≥100 mg/dL	55.4% (17,129)	53.2% (13,558)	65.7% (3,571)	
LDL-Ccorr, mg/dL	95.9 (73.9, 121.3)	97.6 (76.1, 122.7)	87.1 (64.4, 114.0)	<0.001
HDL-C, mmol/L	0.96 (0.82, 1.13)	0.96 (0.82, 1.12)	0.97 (0.83, 1.16)	<0.001
Non-HDL-C, mmol/L	3.40 (2.74, 4.18)	3.35 (2.70, 4.13)	3.63 (2.98, 4.43)	<0.001
Lp(a), mg/dL	16.8 (8.9, 36.3)	13.5 (7.9, 23.2)	81.3 (62.4, 107.3)	<0.001
ApoB, mg/dL	83 (70, 99)	82 (68, 98)	89 (75, 106)	<0.001
Triglyceride, mmol/L	1.38 (1.02, 1.93)	1.39 (1.02, 1.96)	1.36 (1.03, 1.81)	<0.001
**Treatment**				
PCI+CABG	79.5% (24,563)	78.7% (20,056)	83.0% (4,507)	<0.001
ACEI/ARB	51.6% (15,952)	51.1% (13,011)	54.1% (2,941)	<0.001
Beta blocker	82.3% (25,443)	82.0% (20,900)	83.6% (4,543)	0.006
Aspirin	93.0% (28,746)	92.8% (23,630)	94.2% (5,116)	<0.001
P2Y12 inhibitor	87.7% (27,103)	87.0% (22,155)	91.1% (4,948)	<0.001

*Values are mean (SD) or median (interquartile range), or % (Number).*

*CKD, chronic kidney disease; AMI, acute myocardial infarction; CHF, congestive heart failure; AF, atrial fibrillation; eGFR, estimated glomerular filtration rate; TC, total cholesterol; LDL-C, low-density-lipoprotein cholesterol; HDL-C, high-density-lipoprotein cholesterol; Lp(a), lipoprotein(a); ApoB, apoprotein B; PCI, percutaneous coronary intervention; CABG, coronary artery bypass graft; ACEI, angiotensin converting enzyme inhibitor; ARB, angiotensin receptor blocker*.

There were 13.2% (4,085), 31.4% (9,694), and 55.4% (17,129) of the patients categorized in 3 LDL-C groups (<70, 70– <100, ≥100 mg/dL), respectively. Correspondingly, the percentages of the patients with Lp(a) ≥ 50 mg/dL were 9.4, 15.2, and 20.9%. Baseline characteristic differences across LDL-C categories and Lp(a) groups by LDL-C categories were shown in Supplementary Tables 2, 3.

### Correlations Between Lp(a) and Other Lipid Measurements

**Figure 2:**
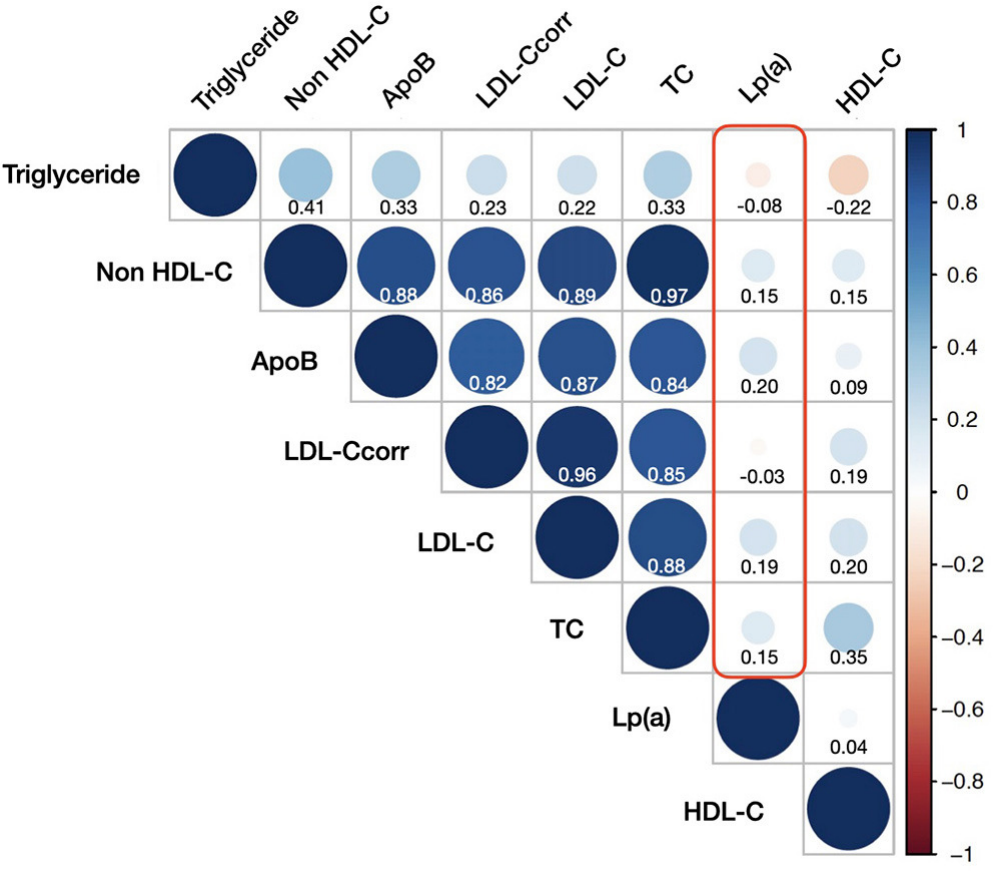
Correlations between lipoprotein(a) and other lipid measurements. HDL-C, high-density-lipoprotein cholesterol; ApoB, apoprotein B; LDL-C_corr_, corrected low-density-lipoprotein cholesterol; LDL-C, low-density-lipoprotein cholesterol; TC, total cholesterol; Lp(a), lipoprotein(a).

Lp(a) showed a significantly positive correlation with LDL-C (*r* = 0.19, *p* <0.001). By contrast, there was a negative and negligible correlation between Lp(a) and LDL-Ccorr (*r* = −0.034, *p* < 0.001). Figure 2 showed all the correlations between Lp(a) and other lipid measurements. In a word, all of the r indexes of Lp(a) associated with other lipid parameters were statistically significant, but their absolute values were ≤ 0.20.

### Kaplan-Meier Survival Curves and Cloglog Tests

**Figure 3:**
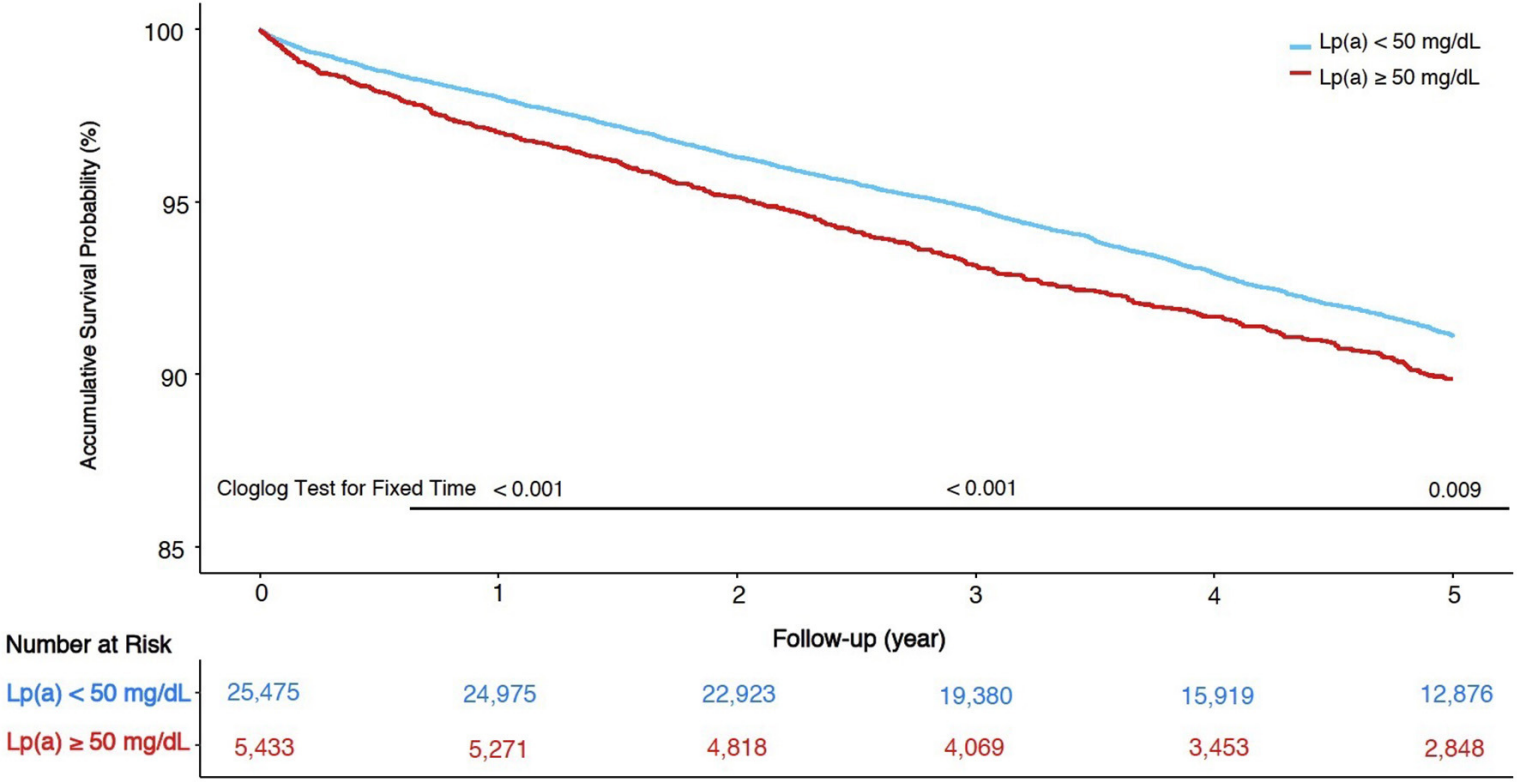
Kaplan-Meier curves of survival and cloglog tests at fixed time points in total cohort.

A total of 664 (2.1%), 1,623 (5.3%), 2,383 (7.7%) patients died in the total cohort at 1-, 3-, and 5-year follow-up. Kaplan-Meier curves of the total cohort for Lp(a) groups were displayed in Figure 3, and the accumulative survival proportions at 1-, 3-, and 5-year follow-up were statistically different between Lp(a) groups (*p*-values of Cloglog test for fixed time points were < 0.001, < 0.001, and 0.009, respectively). Kaplan-Meier curves of each LDL-C category were also shown in Supplementary Figure 1. There were no statistical differences in survival at 1-, 3-, 5-year follow-up between two Lp(a) groups in patients whose LDL-C was <70 mg/dL. Notably, patients with Lp(a) ≥ 50 mg/dL had a lower survival proportion only in those with LDL-C ≥ 100 mg/dL at 5-year follow-up (*p*-value of Cloglog test = 0.017).

### Associations Between Lp(a) and Primary Outcome

In univariate Cox analyses (Supplementary Table 4), patients with Lp(a) ≥ 50 mg/dL had an all-cause death risk HR of 1.18 (95% CI: 1.07–1.31, *p* = 0.003) at 5-year follow-up compared with those with Lp(a) <50 mg/dL in the total cohort. After being adjusted for LDL-C categories, such an association did not change. However, when we analyzed within each LDL-C category, there was no significant association between Lp(a) and all-cause mortality at 5-year follow-up unless the baseline LDL-C was ≥ 100 mg/dL [HR (95% CI, *p*-value) of Lp(a) ≥ 50 mg/dL vs. <50 mg/dL: 1.18 (0.85–1.63, *p* = 0.335) for LDL-C <70 mg/dL; 1.18 (0.98–1.42, *p* = 0.079) for LDL-C ranging from 70 to <100 mg/dL; 1.22 (1.07–1.39, *p* = 0.003) for LDL-C ≥ 100 mg/dL].

**Figure 4:**
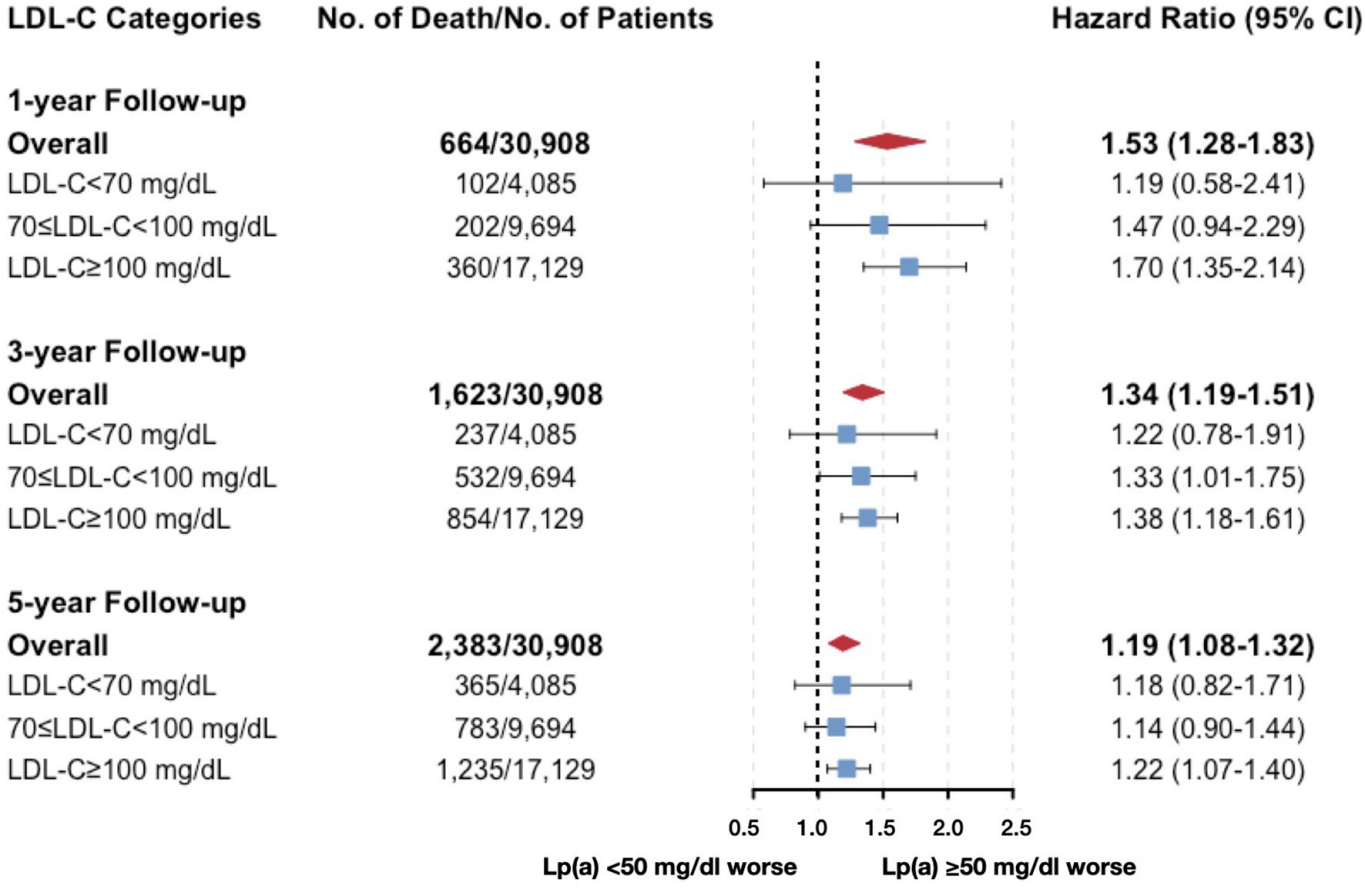
Multivariate cox models evaluating the associations between Lp(a) and all-cause death by LDL-C categories (additionally adjusted for corrected LDL-C). Adjusted for age, gender, congestive heart failure, hypertension, diabetes mellitus, percutaneous coronary intervention or coronary artery bypass graft, estimated glomerular filtration rate, high-density-lipoprotein cholesterol, triglyceride, and corrected LDL-C.

The results of Cox models developed based on the minimal AIC were shown in Supplementary Table 5, which were similar to the results in univariate models. After carefully selection, the multivariate Cox analyses which were fully adjusted for the clinical variables (Table 2) showed that the associations between Lp(a) and all-cause death at 5-year follow-up were robust [HR (95% CI, *p*-value) of Lp(a) ≥ 50 mg/dL vs. <50 mg/dL: 1.19 (1.07–1.31, *p* =0.001) for the total cohort; 1.19 (0.86–1.66, *p* = 0.294) for LDL-C <70 mg/dL; 1.18 (0.98–1.42, *p* = 0.075) for LDL-C ranging from 70 to 100 mg/dL; 1.19 (1.04–1.36, *p* = 0.009) for LDL-C ≥ 100 mg/dL]. Even when we additionally adjusted for LDL-Ccorr, the associations between Lp(a) and 5-year all-cause mortality were unchanged (Figure 4). However, the interaction between Lp(a) groups and LDL-C categories was not statistically significant (*p* = 0.926).

**Table 2:** Multivariate cox regression models for Lp(a) and all-cause mortality.

**Lp(a) ≥50 vs. <50 mg/dL**	**HR**	**95% CI**	***P*-value**	***P* for interaction**
**1-year follow-up**				
Overall	1.50	1.26–1.80	<0.001	
Overall^∧^	1.51	1.26–1.81	<0.001	0.254
LDL-C <70 mg/dL	1.12	0.60–2.11	0.715	
70 ≤ LDL-C <100 mg/dL	1.47	1.05–2.07	0.026	
LDL-C ≥ 100 mg/dL	1.59	1.27–2.00	<0.001	
**3-year follow-up**				
Overall	1.33	1.18–1.50	<0.001	
Overall^∧^	1.33	1.18–1.50	<0.001	0.941
LDL-C <70 mg/dL	1.29	0.87–1.92	0.202	
70 ≤ LDL-C <100 mg/dL	1.36	1.10–1.69	0.005	
LDL-C ≥ 100 mg/dL	1.33	1.14–1.55	<0.001	
**5-year follow-up**				
Overall	1.19	1.07–1.31	0.001	
Overall^∧^	1.19	1.07–1.31	0.001	0.926
LDL-C <70 mg/dL	1.19	0.86–1.66	0.294	
70 ≤ LDL-C <100 mg/dL	1.18	0.98–1.42	0.075	
LDL-C ≥ 100 mg/dL	1.19	1.04–1.36	0.009	

*Adjusted for baseline variables, including age, gender, congestive heart failure, hypertension, diabetes mellitus, percutaneous coronary intervention, or coronary artery bypass graft, estimated glomerular filtration rate, high-density-lipoprotein cholesterol, and triglyceride.*

*^∧^Additionally adjusted for LDL-C categories besides aforementioned variables. When LCL-C was used as a continuous variable, the p for interaction was also insignificant*.

**Figure 5:**
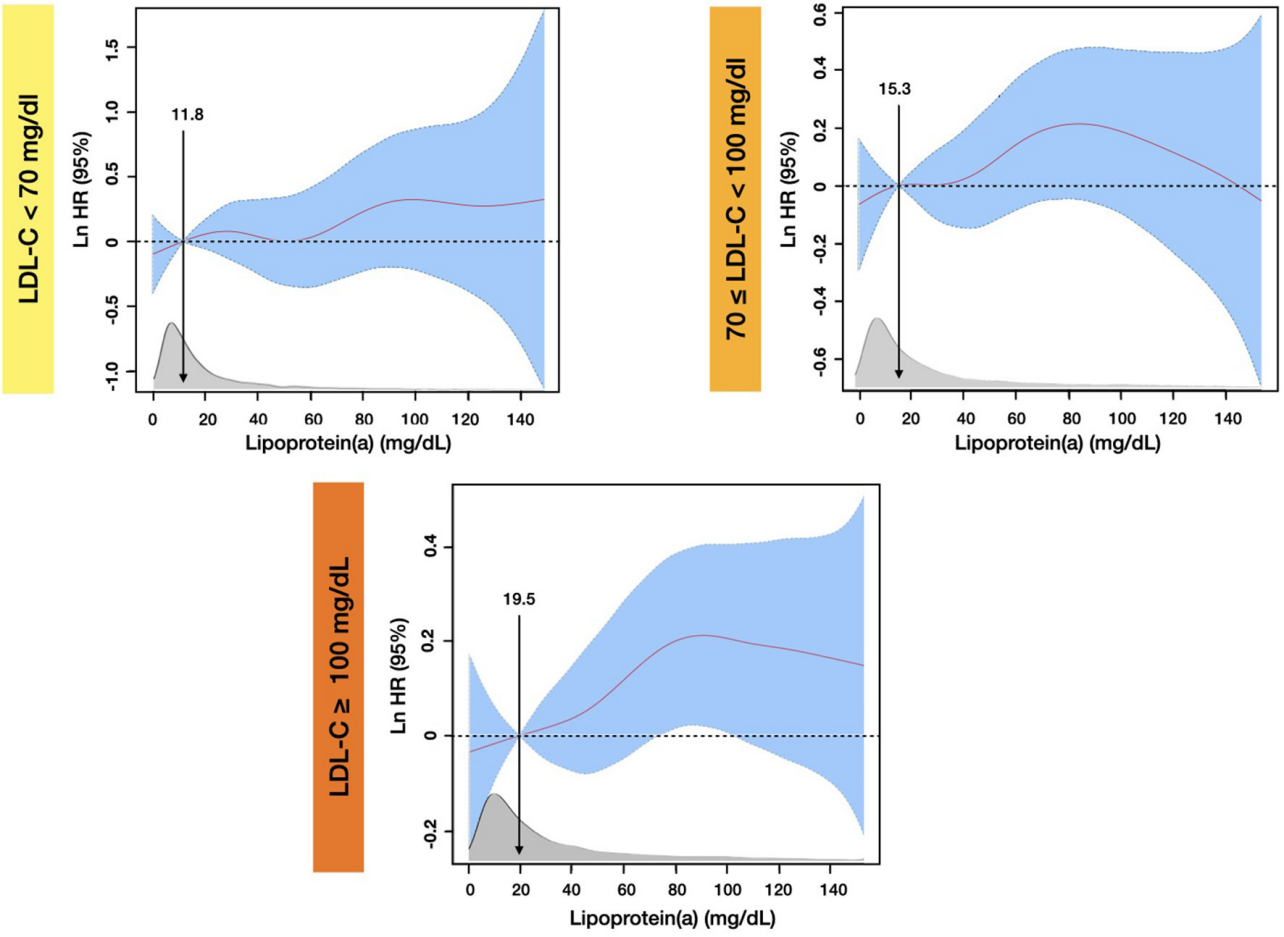
Penalized spline analyses for association between Lp(a) and all-cause mortality by different LDL-C Levels at 5-year follow-up. The penalized spline models were adjusted for age, gender, congestive heart failure, hypertension, diabetes mellitus, percutaneous coronary intervention or coronary artery bypass graft, estimated glomerular filtration rate, high-density-lipoprotein cholesterol, and triglyceride.

Penalized spline models (Figure 5), which were developed based on the same covariates of the multivariate Cox models, showed that compared with the median (19.5 mg/dL) level of Lp(a), the significantly increased risk of all-cause death at 5-year follow-up was observed when Lp(a) was higher than 73 mg/dL in patients with baseline LDL-C ≥ 100 mg/dL. By contrast, there was not any significantly increased all-cause mortality associated with high levels of Lp(a) among patients with LDL-C <100 mg/dL.

### Associations Between Lp(a) and Secondary Outcomes

The results of the associations between Lp(a) and all-cause mortality in the short and medium terms were also displayed in Supplementary Tables 4, 5, Table 2, Figure 4. Most of the results were similar to those at 5-year follow-up, but the results in patients with LDL-C ranging from 70 to <100 mg/dL, Lp(a) ≥ 50 mg/dL were significantly associated with a higher risk of all-cause mortality at 1- and 3-year follow-up. The interaction between Lp(a) groups and baseline LDL-C was not statistically significant. It was noteworthy that the HRs value of Lp(a) within each LDL-C category decreased numerically with the extension of the follow-up time (from 1- to 5-year follow-up).

The results of penalized spline analyses were robust and were displayed in Supplementary Figure 2.

### Sensitivity Analyses

When a threshold of Lp(a) of 30 mg/dL was used, the increased risk of all-cause death associated with Lp(a) ≥ 30 mg/dL was only observed in patients with baseline LDL-C ≥ 100 mg/dL, regardless of the duration of follow-up (Supplementary Tables 6–8). Most results of the sensitivity analyses in patients undergoing PCI or CABG and in patients without AMI were consistent with the results from the total cohort (Supplementary Tables 9–14).

## Discussion

**Figure 6:**
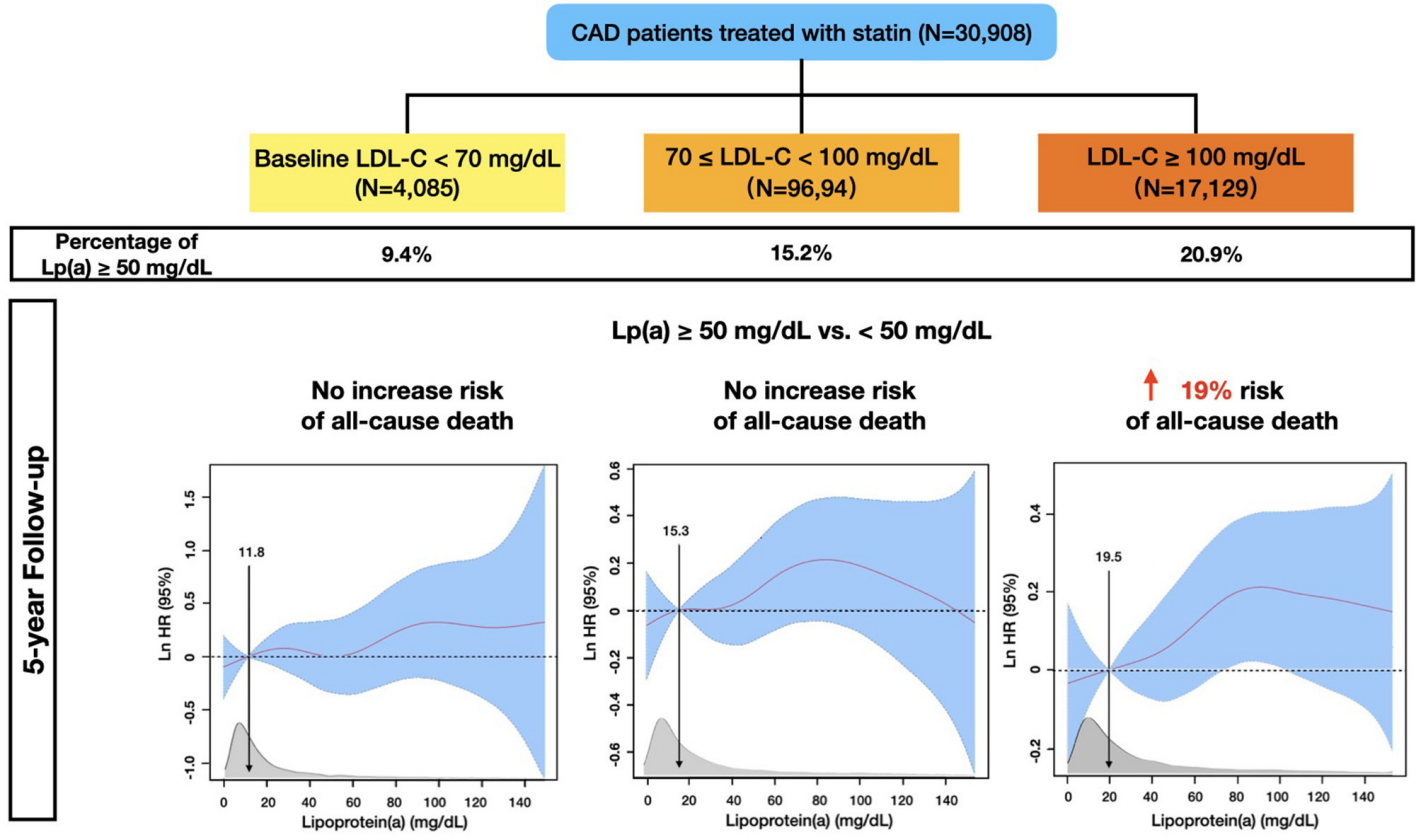
Multivariable-adjusted Risk of All-cause Mortality in 30,908 Statin-treated Coronary Artery Disease Patients from the CIN Registry Study.

Based on this contemporary, large-sample-sized, and statin-treated CAD cohort with a long-term follow-up, our study provides novel insights into the associations between Lp(a) and all-cause mortality across a wide baseline LDL-C spectrum. Interestingly, we found that although Lp(a) ≥ 50 mg/dL appeared to be associated with higher all-cause mortality in CAD patients, such an association attenuated as the baseline levels of LDL-C decreased: Lp(a) ≥ 50 mg/dL was significantly associated with higher all-cause mortality if baseline LDL-C was ≥ 100 mg/dL; by contrast, patients with Lp(a) ≥ 50 mg/dL had a similar risk of all-cause death as those with Lp(a) <50 mg/dL when baseline LDL-C was <70 mg/dL (Figure 6).

Our findings were similar to those in a setting of primary prevention (5, 21). A previous study found that Lp(a)-associated CV risk was significantly increased only in healthy women not taking hormone replacement therapy with LDL-C > 121.4 mg/dL, but not in those with LDL-C <121.4 mg/dL. This finding was similar as what was observed in the study conducted by Verbeek et al. (5). In their study, they found that at LDL-C levels below 2.5 mmol/L (equivalent to 97 mg/dL), the cardiovascular disease risk associated with elevated Lp(a) was attenuated and not statistically significant in a primary preventive population at high risk of cardiovascular diseases (5). Different from these studies, our study focused on a secondary preventive setting, and we found that the increased all-cause mortality associated with Lp(a) > 50 mg/dL was only observed in CAD patients with baseline LDL ≥ 100 mg/dL, but not in those with baseline LDL-C <70 mg/dL. Our findings provided a potential and reasonable explanation for the inconsistent findings of previous studies (11–16).

However, a recent study recruiting 988 ACS patients found that elevated Lp(a) was independently related to adverse prognosis even if their LDL-C at 1-month follow-up was <1.8 mmol/L (equal to 70 mg/dL) (10). Similarly, a *post-hoc* analysis of the ODYDDEY OUTCOMES study indicated that even after LDL-Ccorr was adjusted, a decrease of Lp(a) in alirocumab treatment group was associated with less MACE among ACS patients with a mean LDL-C of 40 mg/dL at 4-month follow-up (9, 22). On the contrary, our study didn't observe any increased risk of all-cause death associated with Lp(a) ≥ 50 mg/dL or ≥ 30 mg/dL in CAD patients with well-controlled LDL-C. A previous meta-analysis also found that the relationship between Lp(a) and MACEs among CAD patients did not achieve statistical significance in studies in which average LDL-C was <130 mg/dL or average total cholesterol was <240 mg/dL (11). Besides, the results of a *post-hoc* analysis of the SATURN study showed that there was no association between higher levels of Lp(a) and the progression of coronary atheroma volume, as well as higher risks of MACEs even if patients' on-treatment LDL-C was <70 mg/dL (16). Reasons for such inconsistent results of the aforementioned studies were still unclear, but one possibility was the differences in the target population (ACS or chronic coronary syndrome). Because of the potential pro-thrombotic property of Lp(a) (6), compared with CAD patients in the stable stage, ACS patients with highly activated inflammatory and pro-thrombotic status and plaque disruption might suffer more from elevated Lp(a) in the early stage even if their LDL-C was well-controlled. Further studies were needed to test this hypothesis through prospective ACS and chronic coronary syndrome cohort with coronary artery structure imaging and serum biomarkers.

To our knowledge, Lp(a) levels in the atherothrombotic range are generally accepted as >30–50 or >75–125 nmol/L (6). The estimated world population with elevated Lp(a) > 50 mg/dL was 1.43 billion, which accounted for around 20% of the global population (23). Our study also found that there were 17.6% of angiography-diagnosed CAD patients whose Lp(a) was ≥ 50 mg/dL. Such a large amount of population but limited healthcare resources required us to identify what kind of individuals would suffer more from elevated Lp(a) and could potentially benefit or benefit more from Lp(a)-lowering treatments. The results of our study indicated that CAD patients with LDL-C ≥ 100 mg/dL might suffer more from elevated Lp(a); by contrast, Lp(a) was not associated with all-cause mortality in patients whose LDL-C was well-controlled. Additionally, among CAD patients with LDL-C levels ranging from 70 to 100 mg/dL, although we didn't observe significant association between Lp(a) ≥ 50 mg/dL and higher all-cause mortality at 5-year follow-up, such an association was significantly positive at short-term and medium-term follow-up. Notably, for the first time, we found that the HRs for Lp(a) within each LDL-C category were numerically decreased as the duration of follow-up was prolonged. The reason is unclear, but this phenomenon suggests the importance of early intervention for elevated Lp(a). These results would be important for risk stratification in clinical practice and helpful to screen appropriate CAD candidates when we conducted randomized clinical trials for Lp(a)-lowering agents in the future.

Similar to a previous study (5, 9), we found that although LDL-C was higher in patients whose Lp(a) was ≥50 mg/dL, LDL-Ccorr was significantly lower. This phenomenon raises our attention that it is necessary to have Lp(a) tests for patients whose LDL-C is difficult to be well-controlled by statins, which would help us to make sure the reasons for statin resistance and to adjust the therapeutic regimen.

Different from statins which might mildly increase Lp(a), some studies found that PCSK9 inhibitors could lower Lp(a) by 20 to 30% (6). The *post-hoc* analyses of the ODYDDEY OUTCOMES study found that among patients with high baseline Lp(a) (≥59.6 mg/dL), the reduction of Lp(a) with alirocumab contributed substantially to the reduction of MACE (9). However, a recent study using the transcriptome analysis showed that the modest Lp(a) lowered by PCSK9 inhibitors did not reduce the pro-inflammatory activation of circulating monocytes (24). It should be emphasized that there were no agents to specifically lower Lp(a) until the invention of liver-targeted antisense oligonucleotide. The phase 2 clinical trials have shown that antisense oligonucleotide pelacarsen reduced mean Lp(a) by 35 to 80% (25). The phase 3 Lp(a)HORIZON [Assessing the Impact of Lipoprotein(a) Lowering with TQJ230 on Major Cardiovascular Events in Patients With CVD) trial] (NCT04023552) is enrolling up to 7,680 patients with established atherosclerotic cardiovascular diseases to evaluate whether pelacarsen will reduce the time to the first occurrence of MACE in patients with baseline Lp(a) ≥ 70 mg/dL or in those with Lp(a) ≥ 90 mg/dL. We are looking forward to its results in 2024.

There are several limitations meriting careful consideration. First, this is a retrospective, observational, and single-centered study so that some inherent bias can not be completely avoided. Secondly, although our study recruited patients with statin treatments, data of the baseline statin dose and history of familial hypercholesterolaemia were unknown, and lipid-lowering treatments as well as the LDL-C levels during follow-up might change. Relevant data were not collected by us so that we failed to evaluate the potential impacts. Thirdly, we used LDL-Ccorr to represent LDL-C which did not contain Lp(a) cholesterol. However, considering somehow inaccuracy of Lp(a) cholesterol estimations by “0.3 ^*^ Lp(a) [mg/dL]” (26), we were not able to evaluate potential effect of such estimations on our results. More accurate estimations for LDL-Ccorr are needed in the future. Additionally, because of the limited number of patients with AMI, we were unable to perform subgroup analyses to assess whether elevated Lp(a) had different effects on the outcomes in different CAD phenotypes. Finally, MACEs were not evaluated in our study due to the lack of relevant data. However, the results of our study with all-cause mortality as the primary outcome also provided critical information to guide our clinical practice.

## Conclusion

In coronary-angiography-diagnosed CAD patients treated with statin therapy, elevated Lp(a) was associated with the increased risks of all-cause death and such an association was modified by the baseline LDL-C. Patients with Lp(a) ≥ 50 mg/dL had higher all-cause mortality compared with those with Lp(a) <50 mg/dL if their baseline LDL-C was ≥100 mg/dL regardless of the duration of follow-up; by contrast, Lp(a) was not associated with all-cause mortality in patients whose LDL-C was <100 mg/dL at 5-year follow-up. These results are important for risk stratification in clinical practice and are helpful to screen appropriate candidates when we conducted randomized clinical trials for Lp(a)-lowering agents in the future. Prospective studies for the impact of Lp(a) on prognosis in CAD patients with different phenotypes are warranted to identify individuals who would benefit or benefit more from Lp(a)-lowering treatments in the future.

## Data Availability Statement

The original contributions presented in the study are included in the article/Supplementary Material, further inquiries can be directed to the corresponding authors.

## Ethics Statement

The studies involving human participants were reviewed and approved by Research Ethics Committee of Guangdong Provincial People's Hospital, Guangdong Academy of Medical Sciences [No. GDREC2019555H(R1)]. Written informed consent for participation was not required for this study in accordance with the national legislation and the institutional requirements.

## Author Contributions

YL, JL, and SC: full access to data and takes responsibility for the integrity and the accuracy of the data analysis. YYao: concept and design. YYan and JD: data management. YYao, BW, ZZ, XL, and ZH: drafting of the manuscript. YL, JC, and NT: critical revision. JL and JC: final approval to publish. All authors acquisition, analysis, and interpretation of data and read and approved the final manuscript.

## Funding

This study was supported by the National Natural Science Foundation of China (Grant Nos. 81670339 and 81970311), Beijing Lisheng Cardiovascular Health Foundation (LHJJ20141751), National Key Research and Development Program of China (Grant No. 2016YFC1301202), and Guangdong Provincial People's Hospital Dengfeng Project Fund (DFJH201919 and DFJH2020026).

## Conflict of Interest

The authors declare that the research was conducted in the absence of any commercial or financial relationships that could be construed as a potential conflict of interest.

## Publisher's Note

All claims expressed in this article are solely those of the authors and do not necessarily represent those of their affiliated organizations, or those of the publisher, the editors and the reviewers. Any product that may be evaluated in this article, or claim that may be made by its manufacturer, is not guaranteed or endorsed by the publisher.
