# Supplementary material for: Baseline Low-Density-Lipoprotein Cholesterol Modifies the Risk of All-Cause Death Associated With Elevated Lipoprotein(a) in Coronary Artery Disease Patients
Source: Front Cardiovasc Med. 2022 Jan 13;8:817442. doi: 10.3389/fcvm.2021.817442 (PMC8792964; doi:10.3389/fcvm.2021.817442)
Supplement: Supplementary file 1 [file Data_Sheet_1.PDF]

Supplementary Materials

1. Imputation for Missing Data

Number and percentage of missing data were displayed in **Supplementary Table 1**. Multiple imputation with chained equation (R package "MICE") was used to deal with missing values. Five imputations were carried out as this has relatively high efficiency. Variables included in the equation were age, gender, acute myocardial infarction, congestive heart failure, atrial fibrillation, stroke, history of revascularization, hemoglobin, estimated glomerular filtration rate, apoprotein B, total cholesterol, low-density-lipoprotein cholesterol, high-density-lipoprotein cholesterol, lipoprotein(a), triglyceride, ACEI/ARB, beta blocker, aspirin, P2Y12 inhibitor, along with the Nelson-Aalen estimator of the baseline cumulative hazard, and the indicator of all-cause death at 5-year follow-up.

2. Supplementary Materials for Statistical Analysis

The differences of baseline characteristics were also evaluated across LDL-C groups. Continuous variables were compared by the one-way ANOVA or Kruskal-Wallis rank test as appropriate, and categorized variables were assessed by Chi-square test.

In choosing covariables for multivariate Cox regression models, we used a backward stepwise method according to minimal Akaike information criterion (AIC), with all baseline variables entered to identify variables associated with all-cause death. Subsequently, an intersection of variables in all cohort and three LDL-C groups for each follow-up time point would be determined. And then full models (for the total cohort and each LDL-C categories) with all variables of this intersection was built. Based on the limited number of events (n=102) in the LDL-C < 70 mg/dL group at 1-year follow-up, a maximal number of variables allowed to be introduced into our models was 10 (according to the event-per-variable principal which indicated that adding an additional variable needed at least 10 events). Therefore, variables were carefully chosen by us based on their values of ($\chi 2$ - df) in full models, covariates in previous studies [1-8], and other considerations (eg, availability of variables in our database). In the end, the following covariates were included: age, gender, congestive heart failure, hypertension, diabetes mellitus, percutaneous coronary intervention or coronary artery bypass graft, estimated glomerular filtration rate, high-density-lipoprotein cholesterol, and triglyceride.

Reference

1. Bittner VA, Szarek M, Aylward PE, et al. Effect of Alirocumab on Lipoprotein(a) and Cardiovascular Risk After Acute Coronary Syndrome. J Am Coll Cardiol, 2020, 75: 133-144.

2. Ren YK, Pan WL, Li XS, et al. The Predictive Value of Lp(a) for Adverse Cardiovascular Event in ACS Patients With an Achieved LDL-C Target at Follow Up After PCI. J Cardiovasc Transl Res, 2021, undefined: undefined.

3. O' Donoghue ML, Morrow DA, Tsimikas S, et al. Lipoprotein(a) for risk assessment in patients with established coronary artery disease. J Am Coll Cardiol 2014;63:520–7.

4. Jin JL, Cao YX, Zhang HW, et al. Lipoprotein(a) and Cardiovascular Outcomes in Patients With Coronary Artery Disease and Prediabetes or Diabetes. Diabetes Care, 2019, 42: 1312-1318.

5. Schwartz GG, Ballantyne CM, Barter PJ, et al. Association of lipoprotein(a) with risk of recurrent ischemic events following acute coronary syndrome: analysis of the dal-Outcomes randomized clinical trial. JAMA Cardiol 2018;3:164–8.

6. Gencer B, Rigamonti F, Nanchen D, et al. Prognostic value of elevated lipoprotein(a) in patients with acute coronary syndromes. Eur J Clin Invest, 2019, 49: e13117.

7. Nestel PJ, Barnes EH, Tonkin AM, et al. Plasma lipoprotein(a) concentration predicts future coronary and cardiovascular events in patients with stable coronary heart disease. Arteriscler Thromb Vasc Biol 2013;33(12):2902-8.

8. Puri R, Ballantyne CM, Hoogeveen RC, et al. Lipoprotein(a) and coronary atheroma progression rates during long-term high-intensity statin therapy: Insights from SATURN. Atherosclerosis. 2017,263:137-144.

3. Supplementary Tables and Figures

Supplementary Table 1. Missing Values of Baseline Variables

	Missing Number (Percentage)
**Demographics**	
Age	0 (0)
Male	0 (0)
**Comorbidities**	
Hypertension	0 (0)
Diabetes	0 (0)
AMI	0 (0)
CHF	0 (0)
Stroke	0 (0)
AF	0 (0)
**Laboratory Measurements**	
Hemoglobin	330 (1.07%)
eGFR	0 (0)
TC	27 (0.09%)
LDL-C	0 (0)
HDL-C	0 (0)
Lp(a)	0 (0)
ApoB	8 (0.03%)
TRIG	27 (0.09%)
**Treatments**	
PCI+CABG	0 (0)
ACEI/ARB	0 (0)
Beta blocker	0 (0)
Aspirin	0 (0)
P2Y12 inhibitor	0 (0)

CKD, chronic kidney disease; AMI, acute myocardial infarction; CHF, congestive heart failure; AF, atrial fibrillation; eGFR, estimated glomerular filtration rate; TC, total cholesterol; LDL-C, low-density-lipoprotein cholesterol; HDL-C, high-density-lipoprotein cholesterol; Lp(a), lipoprotein(a); ApoB, apoprotein B; PCI, percutaneous coronary intervention; CABG, coronary artery bypass graft; ACEI, angiotensin converting enzyme inhibitor; ARB, angiotensin receptor blocker. CKD, LDL-C_{corr}, LDL-C categories, and non-HDL-C were computed based on other baseline variables.

Supplementary Table 2. Baseline Characteristics of Patients by LDL-C Groups

**LDL-C Categories**	**< 70 mg/dL**	**70-<100 mg/dL**	**≥ 100 mg/dL**	**P value**
**Demographics**				
Age, years	64.8±10.9	63.7±10.5	62.4±10.6	< 0.001
Male, (%)	78.8%	78.2%	75.4%	< 0.001
**Comorbidities**				
Hypertension, (%)	62.2%	58.2%	54.9%	< 0.001
Diabetes, (%)	33.4%	29.1%	25.8%	< 0.001
CKD, (%)	21.2%	19.2%	17.7%	< 0.001
AMI, (%)	14.1%	19.6%	24.3%	< 0.001
CHF, (%)	7.7%	8.7%	9.7%	< 0.001
Stroke, (%)	6.8%	6.6%	5.2%	< 0.001
AF, (%)	3.7%	2.7%	2.6%	0.001
**Laboratory measurement**				
Hemoglobin, g/L	130±17	133±16	136±16	< 0.001
eGFR, ml/min/1.73m^2	78.3±23.0	79.1±22.0	80.5±22.4	< 0.001
TC, mmol/L	3.08 (2.77, 3.46)	3.80 (3.49, 4.12)	5.06 (4.53, 5.73)	< 0.001
LDL-C, mg/dL	59.9 (51.8, 65.7)	86.2 (78.9, 93.2)	126.8 (112.1, 147.3)	< 0.001
LDL-C_{corr}, mg/dL	53.7 (44.8, 60.6)	79.0 (70.8, 86.9	118.0 (103.6, 138.7)	< 0.001
HDL-C, mmol/L	0.89 (0.75, 1.07)	0.93 (0.79, 1.09)	0.99 (0.85, 1.16)	< 0.001
Non-HDL-C, mmol/L	2.13 (1.88, 2.45)	2.82 (2.55, 3.12)	4.03 (3.55, 4.68)	< 0.001
Lp(a), mg/dL	11.8 (6.5, 23.4)	15.3 (8.3, 31.9)	19.5 (10.3, 41.9)	< 0.001
Lp(a) ≥ 30 mg/dL, (%)	18.8%	26.7%	34.7%	< 0.001
Lp(a) ≥ 50 mg/dL, (%)	9.4%	15.2%	20.9%	< 0.001
ApoB, mg/dL	57 (51, 64)	72 (66, 79)	97 (86, 110)	< 0.001
TRIG, mmol/L	1.13 (0.83, 1.68)	1.28 (0.95, 1.78)	1.49 (1.12, 2.04)	< 0.001
**Treatment**				
PCI+CABG, (%)	78.5%	79.3%	79.8%	0.142
ACEI/ARB, (%)	48.5%	51.6%	52.4%	< 0.001
Beta blocker, (%)	81.8%	82.3%	82.5%	0.578
Aspirin, (%)	91.2%	92.7%	93.6%	< 0.001
P2Y12 inhibitor, (%)	86.7%	87.2%	88.2%	0.004

Values are mean (SD) or median (interquartile range), or %.

CKD, chronic kidney disease; AMI, acute myocardial infarction; CHF, congestive heart failure; AF, atrial fibrillation; eGFR, estimated glomerular filtration rate; TC, total cholesterol; LDL-C, low-density-lipoprotein cholesterol; HDL-C, high-density-lipoprotein cholesterol; Lp(a), lipoprotein(a); ApoB, apoprotein B; PCI, percutaneous coronary intervention; CABG, coronary artery bypass graft; ACEI, angiotensin converting enzyme inhibitor; ARB, angiotensin receptor blocker.

Supplementary Table 3. Baseline Characteristics of Patients by Levels of Lp(a) in each LDL-C Group

LDL-C (mg/dL)	LDL-C < 70 mg/dL		70 ≤ LDL-C < 100 mg/dL		LDL-C ≥ 100 mg/dL	
Lp(a) (mg/dL)	< 50 mg/dL (N=3,700)	≥ 50 mg/dL (N=385)	< 50 mg/dL (N=8,217)	≥ 50 mg/dL (N=1,477)	< 50 mg/dL (N=13,558)	≥ 50 mg/dL (N=3,571)
**Demographics**						
Age, years	64.9 (10.8)	64.1 (11.5)	63.8 (10.5)	63.4 (10.3)	62.4 (10.7)	62.3 (10.5)
Male, (%)	78.9%	77.1%	78.5%	76.6%	75.9%	73.4%
**Comorbidities**						
Hypertension, (%)	62.0%	64.9%	58.4%	57.1%	55.3%	53.4%
Diabetes, (%)	33.8%	29.4%	29.1%	29.1%	26.5%	23.1%
CKD, (%)	20.7%	26.0%	18.7%	21.8%	16.8%	20.8%
AMI, (%)	14.0%	15.3%	19.6%	20.0%	23.7%	26.3%
CHF, (%)	7.6%	8.6%	8.6%	9.1%	9.3%	11.0%
Stroke, (%)	6.9%	6.0%	6.4%	8.1%	5.0%	5.9%
AF, (%)	3.8%	2.3%	2.9%	1.8%	2.7%	2.1%
**Laboratory Measurement**						
Hemoglobin, g/L	130 (17)	128 (17)	133 (16)	130 (16)	136 (15)	133 (16)
eGFR, ml/min/1.73m2	78.4 (22.9)	77.6 (24.2)	79.4 (22.4)	77.9 (22.6)	80.9 (22.2)	78.6 (22.9)
TC, mmol/L	3.07 (2.76, 3.46)	3.15 (2.86, 3.48)	3.79 (3.48, 4.11)	3.80 (3.50, 4.15)	5.03 (4.51, 5.70)	5.13 (4.60-5.87)
LDL-C, mg/dL	59.9 (51.6, 65.4)	61.9 (54.9, 67.3)	86.2 (78.9, 93.2)	87.8 (80.1, 94.3)	126.1 (111.8, 146.2)	130.3 (114.1, 153.9)
LDL-Ccorr, mg/dL	55.1 (47.0, 61.2)	38.1 (29.3, 45.5)	81.3 (73.8, 88.2)	61.7 (53.4, 69.5)	120.6 (106.7, 140.8)	104.3 (87.8, 127.2)
HDL-C, mmol/L	0.89 (0.75, 1.07)	0.92 (0.77, 1.09)	0.93 (0.79, 1.09)	0.93 (0.80, 1.11)	0.99 (0.85, 1.15)	1.00 (0.86, 1.17)
Non-HDL-C, mmol/L	2.13 (1.87, 2.45)	2.19 (1.98, 2.44)	2.82 (2.55, 3.12)	2.84 (2.58, 3.11)	4.01 (3.53, 4.65)	4.11 (3.60, 4.79)
Lp(a), mg/dL	10.7 (6.2, 18.4)	71.4 (58.2, 92.0)	12.5 (7.5, 22.3)	79.0 (61.6, 103.8)	14.9 (8.9, 24.9)	83.9 (63.4, 110.6)
Lp(a) ≥ 30 mg/dL, (%)	57 (50, 64)	60 (53, 66)	72 (66, 79)	74 (67, 81)	96 (86, 109)	100 (88, 114)
Lp(a) ≥ 50 mg/dL, (%)	1.14 (0.84, 1.70)	1.06 (0.79, 1.45)	1.29 (0.95, 1.81)	1.23 (0.91, 1.64)	1.51 (1.12, 2.08)	1.44 (1.11, 1.90)
ApoB, mg/dL	78.2%	80.5%	78.4%	84.4%	79.1%	82.6%
TRIG, mmol/L	48.0%	53.0%	51.2%	53.8%	51.9%	54.4%
**Treatment**						
PCI+CABG, (%)	81.5%	84.9%	82.2%	82.9%	82.1%	83.8%
ACEI/ARB, (%)	91.0%	93.3%	92.6%	93.4%	93.3%	94.6%
Beta blocker, (%)	86.5%	88.3%	86.4%	91.3%	87.4%	91.3%

Values are mean (SD) or median (interquartile range), or % (Number).

CKD, chronic kidney disease; AMI, acute myocardial infarction; CHF, congestive heart failure; AF, atrial fibrillation; eGFR, estimated glomerular filtration rate; TC, total cholesterol; LDL-C, low-density-lipoprotein cholesterol; HDL-C, high-density-lipoprotein cholesterol; Lp(a), lipoprotein(a); ApoB, apoprotein B; PCI, percutaneous coronary intervention; CABG, coronary artery bypass graft; ACEI, angiotensin converting enzyme inhibitor; ARB,

angiotensin receptor blocker.

Yellow background indicated that the p value for the difference between Lp(a) groups was < 0.05.

Supplementary Table 4. Univariate Cox Regression Models for Lp(a) (< 50 vs. ≥50 mg/dL) and All-cause Mortality

**Lp(a)**<br>**≥ 50 vs. < 50 mg/dL**	**HR**	**95% CI**	**P value**	**P for Interaction**
**1-year Follow-up**				
Overall	1.52	1.27-1.82	< 0.001	
Overall^	1.55	1.30-1.85	< 0.001	0.216
LDL-C < 70 mg/dL	1.16	0.62-2.18	0.635	
70 ≤ LDL-C <100 mg/dL	1.47	1.05-2.06	0.027	
LDL-C ≥100 mg/dL	1.66	1.32-2.08	< 0.001	
**3-year Follow-up**				
Overall	1.33	1.18-1.50	< 0.001	
Overall^	1.36	1.20-1.53	< 0.001	0.842
LDL-C < 70 mg/dL	1.29	0.87-1.92	0.201	
70 ≤ LDL-C <100 mg/dL	1.35	1.09-1.67	0.006	
LDL-C ≥100 mg/dL	1.37	1.18-1.59	< 0.001	
**5-year Follow-up**				
Overall	1.18	1.07-1.31	0.001	
Overall^	1.20	1.09-1.33	< 0.001	0.820
LDL-C < 70 mg/dL	1.18	0.85-1.63	0.335	
70 ≤ LDL-C <100 mg/dL	1.18	0.98-1.42	0.079	
LDL-C ≥ 100 mg/dL	1.22	1.07-1.39	0.003	

^ Additionally adjusted for LDL-C categories.

Supplementary Table 5. Multivariate Cox Regression Models for Lp(a) (< 50 vs. ≥50 mg/dL) and All-cause Mortality based on Akaike Information Criterion

**Lp(a)**<br>**≥ 50 vs. < 50 mg/dL**	**HR**	**95% CI**	**P value**
**1-year Follow-up**			
Overall[#]	1.44	1.20-1.73	< 0.001
LDL-C < 70 mg/dL[#1]	1.12	0.60-2.10	0.722
70 ≤ LDL-C <100 mg/dL[#2]	1.41	1.00-1.99	0.047
LDL-C ≥ 100 mg/dL[#3]	1.51	1.20-1.90	< 0.001
**3-year Follow-up**			
Overall*	1.32	1.17-1.49	< 0.001
LDL-C < 70 mg/dL*	1.29	0.87-1.92	0.209
70 ≤ LDL-C <100 mg/dL*[2]	1.34	1.08-1.66	0.008
LDL-C ≥ 100 mg/dL*[3]	1.37	1.17-1.60	< 0.001
**5-year Follow-up**			
Overall^	1.18	1.06-1.30	0.001
LDL-C < 70 mg/dL^	1.21	0.87-1.68	0.256
70 ≤ LDL-C <100 mg/dL^[2]	1.17	0.97-1.40	0.107
LDL-C ≥ 100 mg/dL^[3]	1.21	1.06-1.38	0.006

Adjusted for age, gender, congestive heart failure, hypertension, diabetes, atrial fibrillation, stroke, percutaneous coronary intervention or coronary artery bypass graft, hemoglobin, eGFR, total cholesterol, HDL-C, triglyceride, and aspirin.

#1 Adjusted for congestive heart failure, eGFR, and HDL-C.

#2 Adjusted for age, gender, congestive heart failure, hypertension, diabetes, atrial fibrillation, percutaneous coronary intervention or coronary artery bypass graft, hemoglobin, and eGFR.

#3 Adjusted for age, gender, acute myocardial infarction, congestive heart failure, hypertension, diabetes, stroke, percutaneous coronary intervention or coronary artery bypass graft, hemoglobin, eGFR, total cholesterol, HDL-C, triglyceride, and aspirin.

*Adjusted for age, gender, acute myocardial infarction, congestive heart failure, hypertension, diabetes, atrial fibrillation, stroke, percutaneous coronary intervention or coronary artery bypass graft, hemoglobin, eGFR, HDL-C, triglyceride, and aspirin.

*1 Adjusted for age, congestive heart failure, hypertension, atrial fibrillation, percutaneous coronary intervention or coronary artery bypass graft, eGFR, HDL-C, and triglyceride.

*2 Adjusted for age, gender, congestive heart failure, atrial fibrillation, percutaneous coronary intervention or coronary artery bypass graft, and hemoglobin.

*3 Adjusted for age, gender, acute myocardial infarction, congestive heart failure, hypertension, diabetes, stroke, percutaneous coronary intervention or coronary artery bypass graft, eGFR, HDL-C, triglyceride, corrected LDL-C, aspirin, and P2Y12 inhibitor.

^ Adjusted for age, gender, congestive heart failure, hypertension, diabetes, atrial fibrillation, stroke, percutaneous coronary intervention or coronary artery bypass graft, hemoglobin, eGFR, HDL-C, triglyceride, and aspirin.

^1 Adjusted for age, gender, congestive heart failure, atrial fibrillation, eGFR, HDL-C, and aspirin.

^2 Adjusted for age, gender, congestive heart failure, atrial fibrillation, stroke, percutaneous coronary intervention or coronary artery bypass graft, hemoglobin, eGFR, total cholesterol, HDL-C, and beta blocker.

^3 Adjusted for age, gender, acute myocardial infarction, congestive heart failure, diabetes, stroke, percutaneous coronary intervention or coronary artery bypass graft, hemoglobin, eGFR, HDL-C, triglyceride, corrected LDL-C, aspirin, and P2Y12 inhibitor.

Supplementary Table 6. Multivariate Cox Regression Models for Lp(a) and All-cause Mortality (additionally adjusted for LDL-C_{corr})

**Lp(a)<br>≥ 50 vs. < 50 mg/dL**	**HR**	**95% CI**	**P value**
**1-year Follow-up**			
Overall	1.53	1.28-1.83	< 0.001
LDL-C < 70 mg/dL	1.19	0.58-2.41	0.636
70 ≤ LDL-C <100 mg/dL	1.47	0.94-2.29	0.09
LDL-C ≥ 100 mg/dL	1.70	1.35-2.14	< 0.001
**3-year Follow-up**			
Overall	1.34	1.19-1.51	< 0.001
LDL-C < 70 mg/dL	1.22	0.78-1.91	0.382
70 ≤ LDL-C <100 mg/dL	1.33	1.01-1.75	0.048
LDL-C ≥100 mg/dL	1.38	1.18-1.61	< 0.001
**5-year Follow-up**			
Overall	1.19	1.08-1.32	0.001
LDL-C < 70 mg/dL	1.18	0.82-1.71	0.371
70 ≤ LDL-C <100 mg/dL	1.14	0.90-1.44	0.287
LDL-C ≥100 mg/dL	1.22	1.07-1.40	0.003

Adjusted for age, gender, congestive heart failure, hypertension, diabetes mellitus, percutaneous coronary intervention or coronary artery bypass graft, estimated glomerular filtration rate, high-density-lipoprotein cholesterol, triglyceride, and corrected LDL-C.

Supplementary Figure 1. Kaplan-Meier Curves of Survival and Cloglog Tests at Fixed Time Points by baseline LDL-C levels

(A) Baseline LDL-C < 70 mg/dL

(B) 70 ≤ Baseline LDL-C < 100 mg/dL

(C) Baseline LDL-C ≥ 100 mg/dL

A. Baseline LDL-C < 70 mg/dL

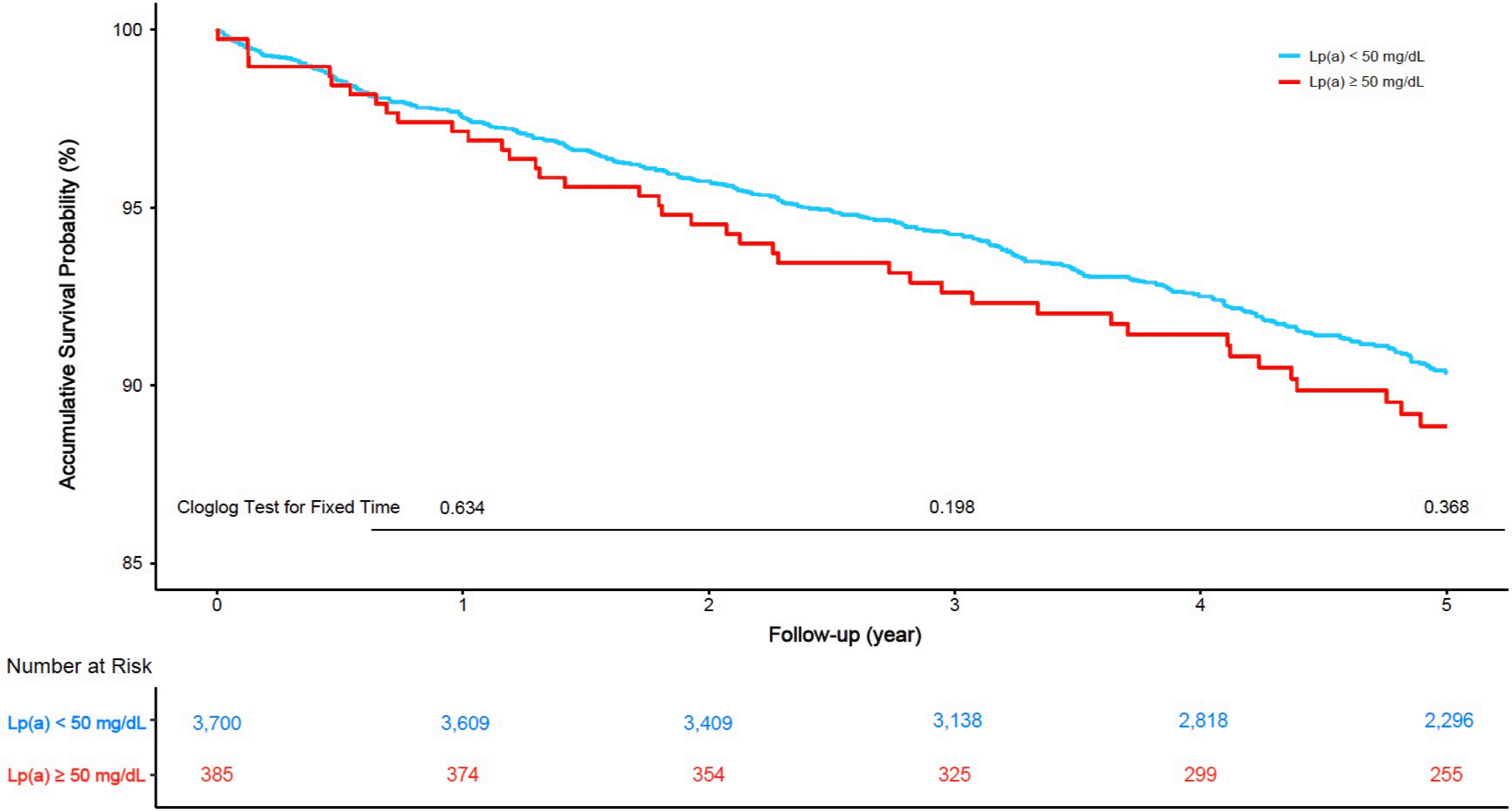

B. 70 < Baseline LDL-C ≤ 100 mg/dL

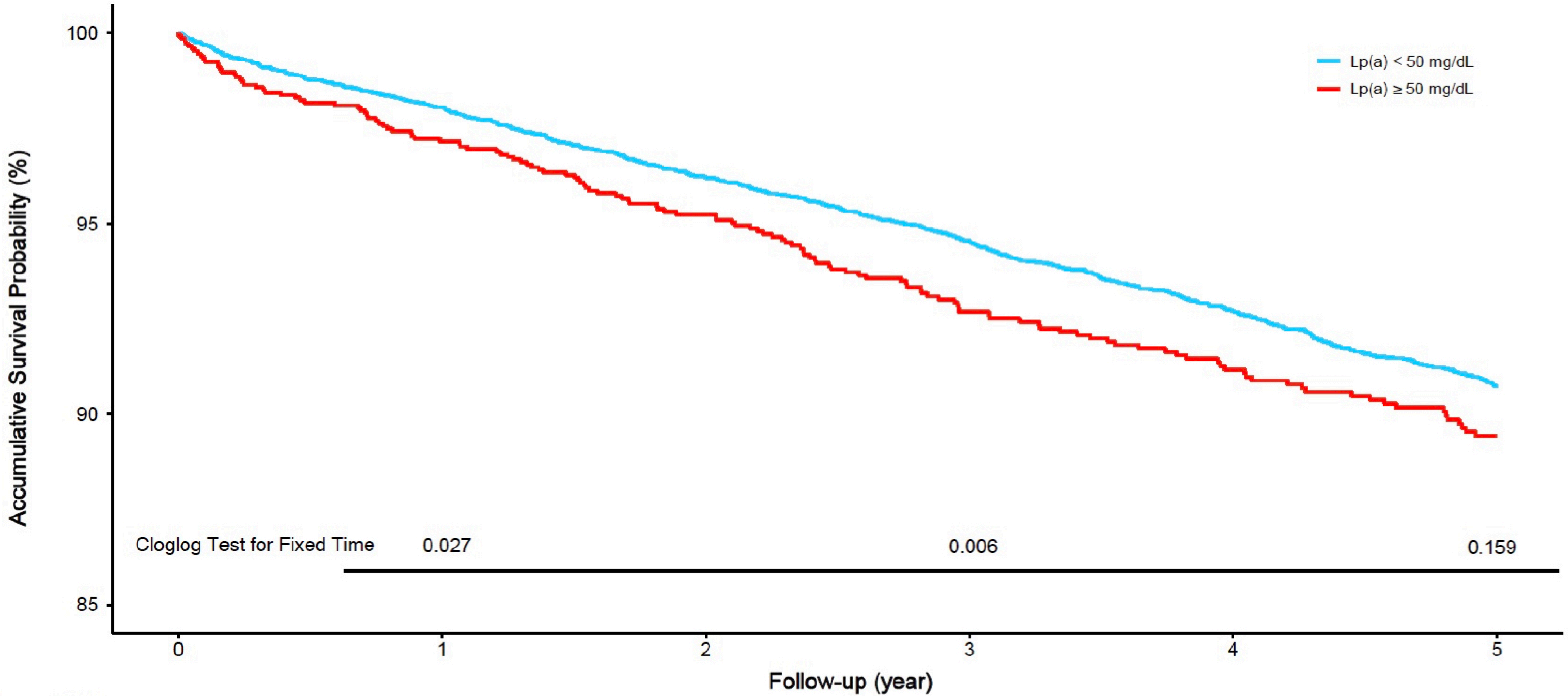

Number at Risk

	0	1	2	3	4	5
Lp(a) < 50 mg/dL	8,217	8,057	7,425	6,288	5,203	4,233
Lp(a) ≥ 50 mg/dL	1,477	1,435	1,310	1,113	954	820

C. Baseline LDL-C ≥ 100 mg/dL

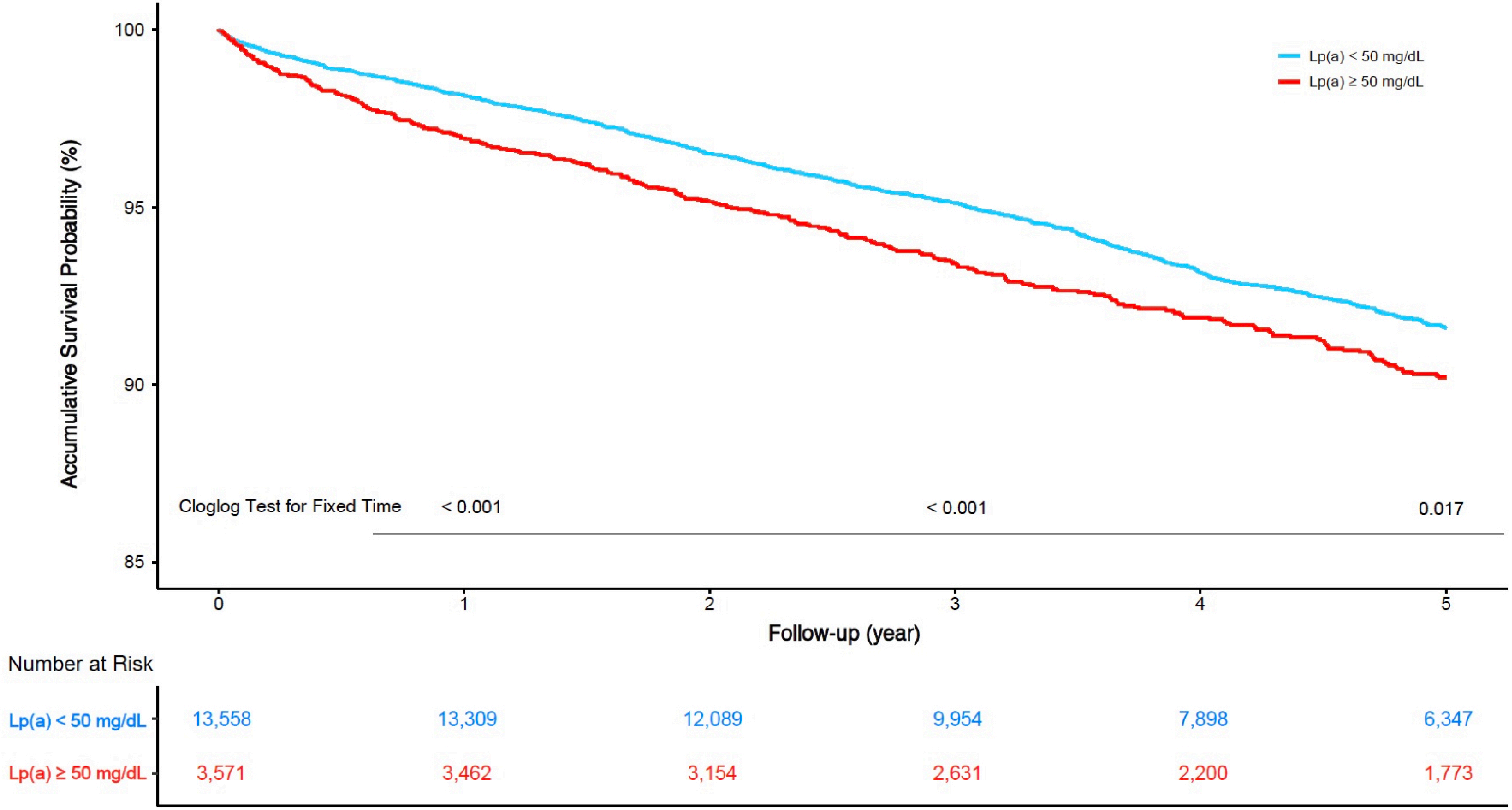

Supplementary Figure 2. Penalized Spline Analyses for Association between Lp(a) and All-cause Mortality by LDL-C Categories

(A) At 1-year Follow-up

(B) At 3-year Follow-up

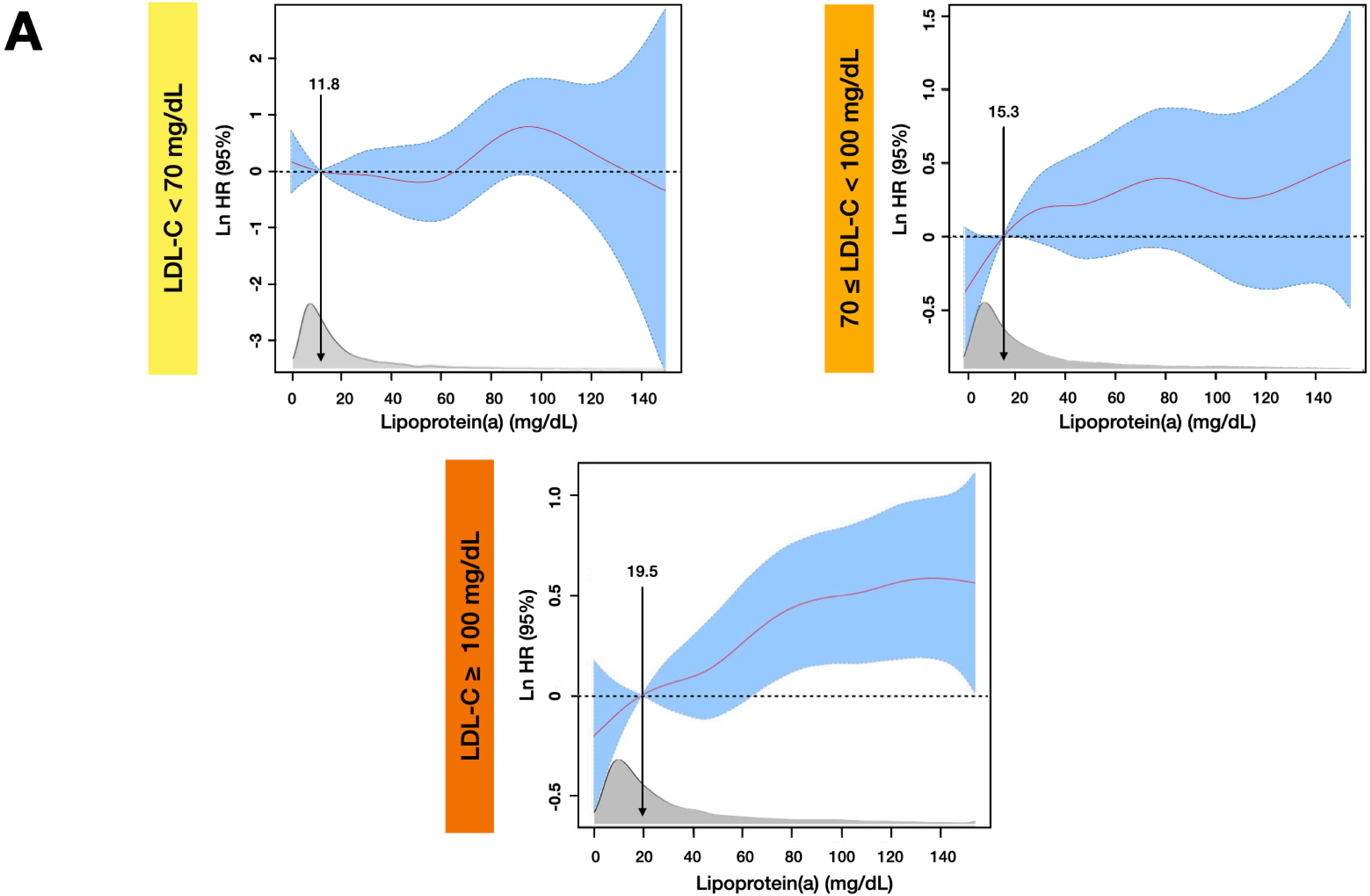

A
LDL-C < 70 mg/dL
Ln HR (95%)
11.8
Lipoprotein(a) (mg/dL)
70 ≤ LDL-C < 100 mg/dL
Ln HR (95%)
15.3
Lipoprotein(a) (mg/dL)
LDL-C ≥ 100 mg/dL
Ln HR (95%)
19.5
Lipoprotein(a) (mg/dL)

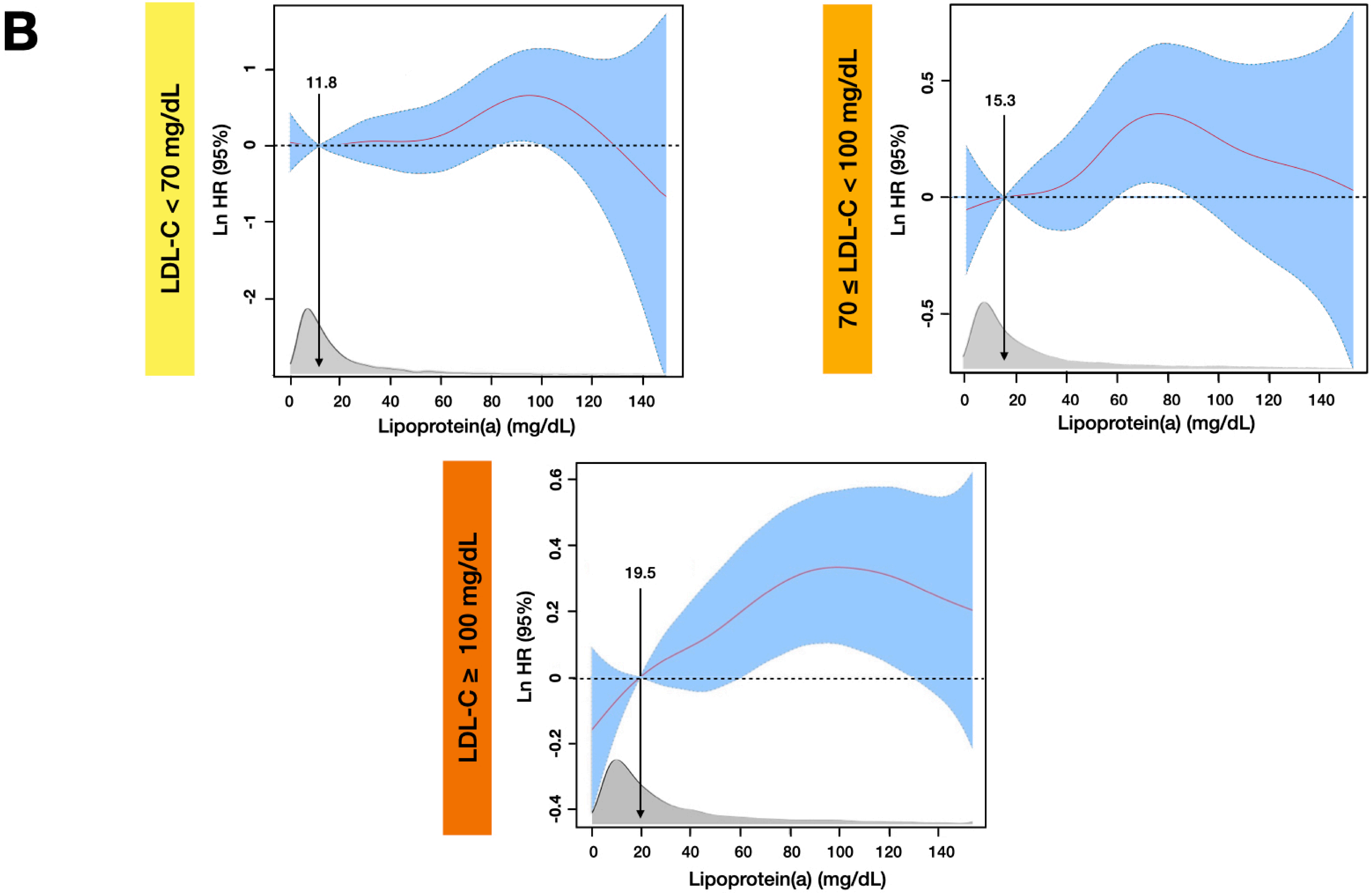

B
LDL-C < 70 mg/dL
Ln HR (95%)
1
0
-1
-2
11.8
0
20
40
60
80
100
120
140
Lipoprotein(a) (mg/dL)
70 ≤ LDL-C < 100 mg/dL
Ln HR (95%)
0.5
0
-0.5
15.3
0
20
40
60
80
100
120
140
Lipoprotein(a) (mg/dL)
LDL-C ≥ 100 mg/dL
Ln HR (95%)
0.6
0.4
0.2
0
-0.2
-0.4
19.5
0
20
40
60
80
100
120
140
Lipoprotein(a) (mg/dL)

Results for Sensitivity Analyses

Supplementary Table 7. Univariate Cox Regression Models for Lp(a) Categories (< 30 and ≥30 mg/dL) and All-cause Mortality

**Lp(a)**<br>**≥ 30 vs. < 30 mg/dL**	**HR**	**95% CI**	**P value**	**P for Interaction**
**1-year Follow-up**				
Overall	1.36	1.17-1.60	< 0.001	
Overall^	1.39	1.19-1.63	< 0.001	0.399
LDL-C < 70 mg/dL	1.27	0.80-2.01	0.319	
70 ≤ LDL-C <100 mg/dL	1.31	0.97-1.76	0.075	
LDL-C ≥100 mg/dL	1.46	1.19-1.80	< 0.001	
**3-year Follow-up**				
Overall	1.23	1.11-1.37	< 0.001	
Overall^	1.25	1.13-1.39	< 0.001	0.634
LDL-C < 70 mg/dL	1.29	0.95-1.74	0.103	
70 ≤ LDL-C <100 mg/dL	1.16	0.96-1.39	0.124	
LDL-C ≥100 mg/dL	1.31	1.14-1.50	< 0.001	
**5-year Follow-up**				
Overall	1.11	1.02-1.21	0.017	
Overall^	1.13	1.03-1.23	0.006	0.677
LDL-C < 70 mg/dL	1.12	0.87-1.44	0.398	
70 ≤ LDL-C <100 mg/dL	1.08	0.93-1.27	0.303	
LDL-C ≥ 100 mg/dL	1.16	1.03-1.30	0.013	

^ Additionally adjusted for LDL-C categories.

Supplementary Table 8. Multivariate Cox Regression Models for Lp(a) (< 30 and ≥30 mg/dL) and All-cause Mortality

**Lp(a) ≥ 30 vs. < 30 mg/dL**	**HR**	**95% CI**	**P value**	**P for Interaction**
**1-year Follow-up**				
Overall	1.33	1.13-1.55	< 0.001	
Overall^	1.33	1.13-1.56	< 0.001	0.436
LDL-C < 70 mg/dL	1.19	0.74-1.89	0.475	
70 ≤ LDL-C <100 mg/dL	1.30	0.97-1.75	0.081	
LDL-C ≥100 mg/dL	1.37	1.11-1.70	0.003	
**3-year Follow-up**				
Overall	1.21	1.09-1.34	< 0.001	
Overall^	1.21	1.09-1.34	< 0.001	0.635
LDL-C < 70 mg/dL	1.24	0.91-1.68	0.170	
70 ≤ LDL-C <100 mg/dL	1.15	0.95-1.38	0.155	
LDL-C ≥100 mg/dL	1.25	1.09-1.43	0.002	
**5-year Follow-up**				
Overall	1.10	1.01-1.20	0.036	
Overall^	1.10	1.00-1.20	0.039	0.662
LDL-C < 70 mg/dL	1.09	0.85-1.41	0.504	
70 ≤ LDL-C <100 mg/dL	1.07	0.91-1.25	0.401	
LDL-C ≥ 100 mg/dL	1.11	0.99-1.25	0.079	

Adjusted for age, gender, congestive heart failure, hypertension, diabetes mellitus, percutaneous coronary intervention or coronary artery bypass graft, estimated glomerular filtration rate, high-density-lipoprotein cholesterol, and triglyceride.

^ Additionally adjusted for LDL-C categories besides aforementioned variables. When LCL-C was used as a continuous variable, the p for interaction was also insignificant.

Supplementary Table 9. Multivariate Cox Regression Models for Lp(a) (< 30 and ≥30 mg/dL) and All-cause Mortality (additionally adjusted for LDL-C_{corr})

**Lp(a)**<br>**≥ 30 vs. < 30 mg/dL**	**HR**	**95% CI**	**P value**
**1-year Follow-up**			
Overall	1.33	1.14-1.56	< 0.001
LDL-C < 70 mg/dL	1.25	0.74-2.10	0.398
70 ≤ LDL-C <100 mg/dL	1.24	0.87-1.78	0.238
LDL-C ≥ 100 mg/dL	1.43	1.16-1.77	0.001
**3-year Follow-up**			
Overall	1.21	1.09-1.34	< 0.001
LDL-C < 70 mg/dL	1.19	0.85-1.67	0.307
70 ≤ LDL-C <100 mg/dL	1.05	0.84-1.32	0.671
LDL-C ≥100 mg/dL	1.27	1.11-1.46	0.001
**5-year Follow-up**			
Overall	1.10	1.01-1.20	0.035
LDL-C < 70 mg/dL	1.07	0.81-1.42	0.622
70 ≤ LDL-C <100 mg/dL	1.00	0.83-1.21	0.989
LDL-C ≥100 mg/dL	1.13	1.01-1.27	0.046

Adjusted for age, gender, congestive heart failure, hypertension, diabetes mellitus, percutaneous coronary intervention or coronary artery bypass graft, estimated glomerular filtration rate, high-density-lipoprotein cholesterol, triglyceride, and corrected LDL-C.

Supplementary Table 10. Univariate Cox Regression Models for Lp(a) and All-cause Mortality in Patients Undergoing Revascularization

**Lp(a) ≥ 50 vs. < 50 mg/dL**	**HR**	**95% CI**	**P value**	**P for Interaction**
**1-year Follow-up**				
Overall	1.40	1.14-1.72	0.001	
Overall^	1.43	1.16-1.76	0.001	0.273
LDL-C < 70 mg/dL	1.05	0.51-2.19	0.892	
70 ≤ LDL-C <100 mg/dL	1.37	0.93-2.03	0.112	
LDL-C ≥100 mg/dL	1.53	1.18-2.00	0.001	
**3-year Follow-up**				
Overall	1.24	1.09-1.43	0.002	
Overall^	1.27	1.11-1.45	0.001	0.812
LDL-C < 70 mg/dL	1.17	0.73-1.86	0.509	
70 ≤ LDL-C <100 mg/dL	1.28	1.01-1.63	0.052	
LDL-C ≥100 mg/dL	1.28	1.07-1.53	0.006	
**5-year Follow-up**				
Overall	1.12	1.00-1.26	0.043	
Overall^	1.14	1.02-1.28	0.021	0.926
LDL-C < 70 mg/dL	1.14	0.78-1.65	0.504	
70 ≤ LDL-C <100 mg/dL	1.13	0.92-1.39	0.244	
LDL-C ≥ 100 mg/dL	1.15	0.99-1.33	0.061	

^ Additionally adjusted for LDL-C categories.

Supplementary Table 11. Multivariate Cox Regression Models for Lp(a) and All-cause Mortality in Patients Undergoing Revascularization

**Lp(a)**<br>**≥ 50 vs. < 50 mg/dL**	**HR**	**95% CI**	**P value**	**P for Interaction**
**1-year Follow-up**				
Overall	1.37	1.11-1.68	0.003	
Overall^	1.38	1.12-1.70	0.002	0.286
LDL-C < 70 mg/dL	1.05	0.50-2.18	0.900	
70 ≤ LDL-C <100 mg/dL	1.31	0.89-1.94	0.173	
LDL-C ≥100 mg/dL	1.47	1.13-1.92	0.004	
**3-year Follow-up**				
Overall	1.23	1.07-1.41	0.003	
Overall^	1.23	1.07-1.41	0.003	0.895
LDL-C < 70 mg/dL	1.19	0.75-1.91	0.457	
70 ≤ LDL-C <100 mg/dL	1.25	0.97-1.60	0.079	
LDL-C ≥100 mg/dL	1.24	1.04-1.48	0.018	
**5-year Follow-up**				
Overall	1.12	1.00-1.25	0.053	
Overall^	1.12	1.00-1.26	0.054	0.973
LDL-C < 70 mg/dL	1.17	0.80-1.70	0.421	
70 ≤ LDL-C <100 mg/dL	1.11	0.90-1.36	0.347	
LDL-C ≥ 100 mg/dL	1.12	0.97-1.30	0.122	

Adjusted for age, gender, congestive heart failure, hypertension, diabetes mellitus, estimated glomerular filtration rate, high-density-lipoprotein cholesterol, and triglyceride.

^ Additionally adjusted for LDL-C categories besides aforementioned variables. When LCL-C was used as a continuous variable, the p for interaction was also insignificant.

Supplementary Table 12. Multivariate Cox Regression Models for Lp(a) and All-cause Mortality in Patients Undergoing Revascularization (additionally adjusted for LDL-C_{corr})

**Lp(a)** **≥ 50 vs. < 50 mg/dL**	**HR**	**95% CI**	**P value**
**1-year Follow-up**			
Overall	1.39	1.13-1.71	0.002
LDL-C < 70 mg/dL	1.13	0.50-2.56	0.776
70 ≤ LDL-C <100 mg/dL	1.35	0.81-2.25	0.243
LDL-C ≥ 100 mg/dL	1.60	1.22-2.10	0.001
**3-year Follow-up**			
Overall	1.23	1.07-1.41	0.003
LDL-C < 70 mg/dL	1.12	0.66-1.89	0.672
70 ≤ LDL-C <100 mg/dL	1.21	0.88-1.67	0.243
LDL-C ≥ 100 mg/dL	1.28	1.07-1.54	0.008
**5-year Follow-up**			
Overall	1.12	1.00-1.26	0.054
LDL-C < 70 mg/dL	1.18	0.78-1.80	0.433
70 ≤ LDL-C <100 mg/dL	1.03	0.79-1.34	0.842
LDL-C ≥ 100 mg/dL	1.15	0.98-1.34	0.081

Adjusted for age, gender, congestive heart failure, hypertension, diabetes mellitus, estimated glomerular filtration rate, high-density-lipoprotein cholesterol, triglyceride, and corrected LDL-C.

Supplementary Table 13. Univariate Cox Regression Models for Lp(a) and All-cause Mortality in Patients without Acute Myocardial Infarction

**Lp(a)**<br>**≥ 50 vs. < 50 mg/dL**	**HR**	**95% CI**	**P value**	**P for Interaction**
**1-year Follow-up**				
Overall	1.63	1.33-2.01	< 0.001	
Overall^	1.68	1.36-2.07	< 0.001	0.416
LDL-C < 70 mg/dL	1.38	0.71-2.68	0.338	
70 ≤ LDL-C <100 mg/dL	1.59	1.09-2.32	0.015	
LDL-C ≥100 mg/dL	1.79	1.37-2.35	< 0.001	
**3-year Follow-up**				
Overall	1.38	1.21-1.59	< 0.001	
Overall^	1.42	1.23-1.62	< 0.001	0.782
LDL-C < 70 mg/dL	1.40	0.91-2.14	0.126	
70 ≤ LDL-C <100 mg/dL	1.35	1.06-1.72	0.016	
LDL-C ≥100 mg/dL	1.46	1.22-1.74	< 0.001	
**5-year Follow-up**				
Overall	1.19	1.06-1.33	0.003	
Overall^	1.21	1.08-1.36	0.001	0.896
LDL-C < 70 mg/dL	1.29	0.91-1.83	0.146	
70 ≤ LDL-C <100 mg/dL	1.16	0.94-1.43	0.164	
LDL-C ≥ 100 mg/dL	1.23	1.05-1.43	0.009	

^ Additionally adjusted for LDL-C categories.

Supplementary Table 14. Multivariate Cox Regression Models for Lp(a) and All-cause Mortality in Patients without Acute Myocardial Infarction

**Lp(a) ≥ 50 vs. < 50 mg/dL**	**HR**	**95% CI**	**P value**	**P for Interaction**
**1-year Follow-up**				
Overall	1.64	1.33-2.02	< 0.001	
Overall^	1.66	1.35-2.05	< 0.001	0.456
LDL-C < 70 mg/dL	1.28	0.66-2.49	0.468	
70 ≤ LDL-C <100 mg/dL	1.64	1.13-2.40	0.010	
LDL-C ≥100 mg/dL	1.74	1.33-2.29	< 0.001	
**3-year Follow-up**				
Overall	1.40	1.23-1.61	< 0.001	
Overall^	1.41	1.23-1.62	< 0.001	0.853
LDL-C < 70 mg/dL	1.32	0.86-2.02	0.211	
70 ≤ LDL-C <100 mg/dL	1.40	1.10-1.79	0.007	
LDL-C ≥100 mg/dL	1.43	1.19-1.71	< 0.001	
**5-year Follow-up**				
Overall	1.20	1.07-1.35	0.002	
Overall^	1.21	1.08-1.36	0.001	0.765
LDL-C < 70 mg/dL	1.26	0.89-1.79	0.189	
70 ≤ LDL-C <100 mg/dL	1.20	0.97-1.48	0.088	
LDL-C ≥ 100 mg/dL	1.20	1.03-1.40	0.019	

Adjusted for age, gender, congestive heart failure, hypertension, diabetes mellitus, percutaneous coronary intervention or coronary artery bypass graft, estimated glomerular filtration rate, high-density-lipoprotein cholesterol, triglyceride, and corrected LDL-C.

^ Additionally adjusted for LDL-C categories besides aforementioned variables. When LCL-C was used as a continuous variable, the p for interaction was also insignificant.

Supplementary Table 15. Multivariate Cox Regression Models for Lp(a) and All-cause Mortality in Patients without Acute Myocardial Infarction (additionally adjusted for LDL-C_{corr})

**Lp(a)**<br>**≥ 50 vs. < 50 mg/dL**	**HR**	**95% CI**	**P value**
**1-year Follow-up**			
Overall	1.66	1.35-2.04	< 0.001
LDL-C < 70 mg/dL	1.20	0.56-2.58	0.638
70 ≤ LDL-C <100 mg/dL	1.73	1.04-2.86	0.033
LDL-C ≥ 100 mg/dL	1.87	1.42-2.46	< 0.001
**3-year Follow-up**			
Overall	1.40	1.22-1.61	< 0.001
LDL-C < 70 mg/dL	1.11	0.68-1.81	0.682
70 ≤ LDL-C <100 mg/dL	1.34	0.97-1.85	0.072
LDL-C ≥100 mg/dL	1.45	1.21-1.75	< 0.001
**5-year Follow-up**			
Overall	1.20	1.07-1.35	0.002
LDL-C < 70 mg/dL	1.18	0.79-1.75	0.415
70 ≤ LDL-C <100 mg/dL	1.09	0.83-1.43	0.523
LDL-C ≥100 mg/dL	1.24	1.06-1.45	0.008

Adjusted for age, gender, congestive heart failure, hypertension, diabetes mellitus, percutaneous coronary intervention or coronary artery bypass graft, estimated glomerular filtration rate, high-density-lipoprotein cholesterol, triglyceride, and corrected LDL-C.
